# Single‐cell RNA‐sequencing and spatial transcriptomic analysis reveal a distinct population of *APOE* ^−^ cells yielding pathological lymph node metastasis in papillary thyroid cancer

**DOI:** 10.1002/ctm2.70172

**Published:** 2025-01-15

**Authors:** Guohui Xiao, Rongli Xie, Jianhua Gu, Yishu Huang, Min Ding, Dongjie Shen, Jiqi Yan, Jianming Yuan, Qiong Yang, Wen He, Siyu Xiao, Haizhen Chen, Dan Xu, Jian Wu, Jian Fei

**Affiliations:** ^1^ Department of General Surgery Ruijin Hospital Shanghai Jiao Tong University School of Medicine Shanghai China; ^2^ Department of General Surgery Ruijin Hospital Luwan Branch Shanghai Jiao Tong University School of Medicine Shanghai China; ^3^ Department of Thyroid and Breast Surgery Punan Branch of Renji Hospital Shanghai Jiaotong University School of Medicine Shanghai China; ^4^ Department of General Surgery Shanghai Changhang Hospital Shanghai China; ^5^ Department of General Surgery Shanghai International Medical Center Shanghai China; ^6^ Department of Emergency Medicine Ruijin Hospital Shanghai Jiao Tong University School of Medicine Shanghai China; ^7^ Department of Pathology Punan Branch of Renji Hospital Jiaotong University School of Medicine Shanghai China; ^8^ State Key Laboratory of Oncogenes and Related Genes Shanghai China; ^9^ Institute of Translational Medicine Shanghai Jiao Tong University Shanghai China

**Keywords:** APOE‐ABCA1‐LXR axis, lymph node metastasis, papillary thyroid carcinoma, single‐cell RNA‐sequencing

## Abstract

**Background:**

Thyroid cancer is one of the most common endocrine tumors worldwide, especially among women and the metastatic mechanism of papillary thyroid carcinoma remains poorly understood.

**Methods:**

Thyroid cancer tissue samples were obtained for single‐cell RNA‐sequencing and spatial transcriptomics, aiming to intratumoral and antimetastatic heterogeneity of advanced PTC. The functions of *APOE* in PTC cell proliferation and invasion were confirmed through in vivo and in vitro assays. Pseudotime analysis and CellChat were performed to explore the the molecular mechanisms of the *APOE* in PTC progression.

**Results:**

We identified a subpopulation of tumor cells with lower expression levels of *APOE*, associated with advanced stages of PTC and cervical metastasis. *APOE* overexpression significantly reduced tumor cell proliferation and invasion, both in vitro and in vivo, by activating the *ABCA1‐LXR* axis. *APOE^−^* tumor cells may promote tumor growth by interacting with dendritic cells and CD4^+^ T cells via *CD99*‐ rather than CD6‐regulated signaling. We established a machine learning‐based scRNA‐seq data, 13‐gene signature predictive of lymph node metastasis.

**Conclusions:**

We identified a distinct *APOE^−^* tumor cell population associated with cervical metastasis and poor prognosis. Our results and models have potential clinical, prognostic, and therapeutic implications for advanced PTC.

**Key points:**

- A subpopulation of tumor cells with lower expression levels of *APOE* was strongly associated with more advanced stages and metastasis of PTC.
- *APOE*‐negative (*APOE* ^−^) cellsoverall exhibited weaker interactions with immune cells.
- A machine‐learning bioinformatics model based on scRNA‐seq data of in‐situ thyroid cancer tissue was established to predict lymph node metastasis.

## Graphical abstract

We identified a subpopulation of tumor cells with lower expression levels of APOE in both mRNAs and proteins that were highly associated with more advanced stages and metastasis of PTC by integrating single‐cell sequencing and spatial transcriptome assays.

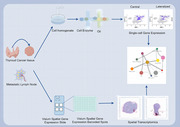

## 1 INTRODUCTION

Thyroid cancer is one of the most common endocrine tumours worldwide, particularly among women.^1^, ^2^ Differentiated thyroid cancer is the most common type, accounting for over 95% of all cases, with papillary thyroid carcinoma (PTC) being the most prevalent subtype. Metastases most commonly involve lymph nodes adjacent to the primary tumour site, while less frequent sites include the lungs and distant bones. Despite a steady increase in incidence, thyroid cancer‐related mortality has remained relatively stable over the past few decades.^2^, ^3^, ^4^, ^5^ Therefore, contemporary challenges for thyroid cancer physicians include avoiding overtreatment, better identifying patients with advanced or high‐risk subtypes and tailoring therapeutic strategies to specific subtypes.

As with other types of tumours, thyroid cancer exhibits a high degree of cellular heterogeneity and is composed of distinct cell types, including neoplastic cells, stromal cells, adipocytes and immune cells.^3^ PTC is reportedly composed of cancer‐associated fibroblasts (CAFs) and lymphocytes that are highly involved in tumour initiation and progression.^6^, ^7^ In PTC, CAFs contribute to tumour volume and expansion, as well as the activation of distinct metabolic pathways, while immune cells exert both positive and negative effects on tumour progression.^6^, ^8^, ^9^, ^10^

Single‐cell RNA sequencing (scRNA‐seq) provides detailed data on individual cell transcriptomes and helps to understand cellular diversity in tumours.^11^ However, it lacks spatial context because tissue must be dissociated before sequencing. Spatial transcriptomics compensates for this limitation by mapping transcripts across entire tissues, albeit at a lower resolution. Combining these two methods provides comprehensive and spatially resolved transcriptional profiles of diverse tumour tissues, improving our understanding of cellular organization and interactions within their natural environment. Various experimental modalities and algorithms have been developed to achieve such integration, including deconvolution and mapping.^12^ Deconvolution is designed to disentangle discrete cellular subpopulations from a single sampling area based on single‐cell data, whereas mapping is designed to create a spatially resolved cell‐type map at single‐cell resolution.^12^

In this study, we aimed to identify specific cell subpopulations and their associated states that are enriched in thyroid tumours and affected lymph node samples. By linking cellular identity and states to their spatial localization within the tumour microenvironment, we aimed to determine the interactions among different cell types within subregions of metastatic PTC.

## 2 RESULTS

### 2.1 Transcriptomic and spatial transcriptomic profiling of thyroid cancer

**FIGURE 1:**
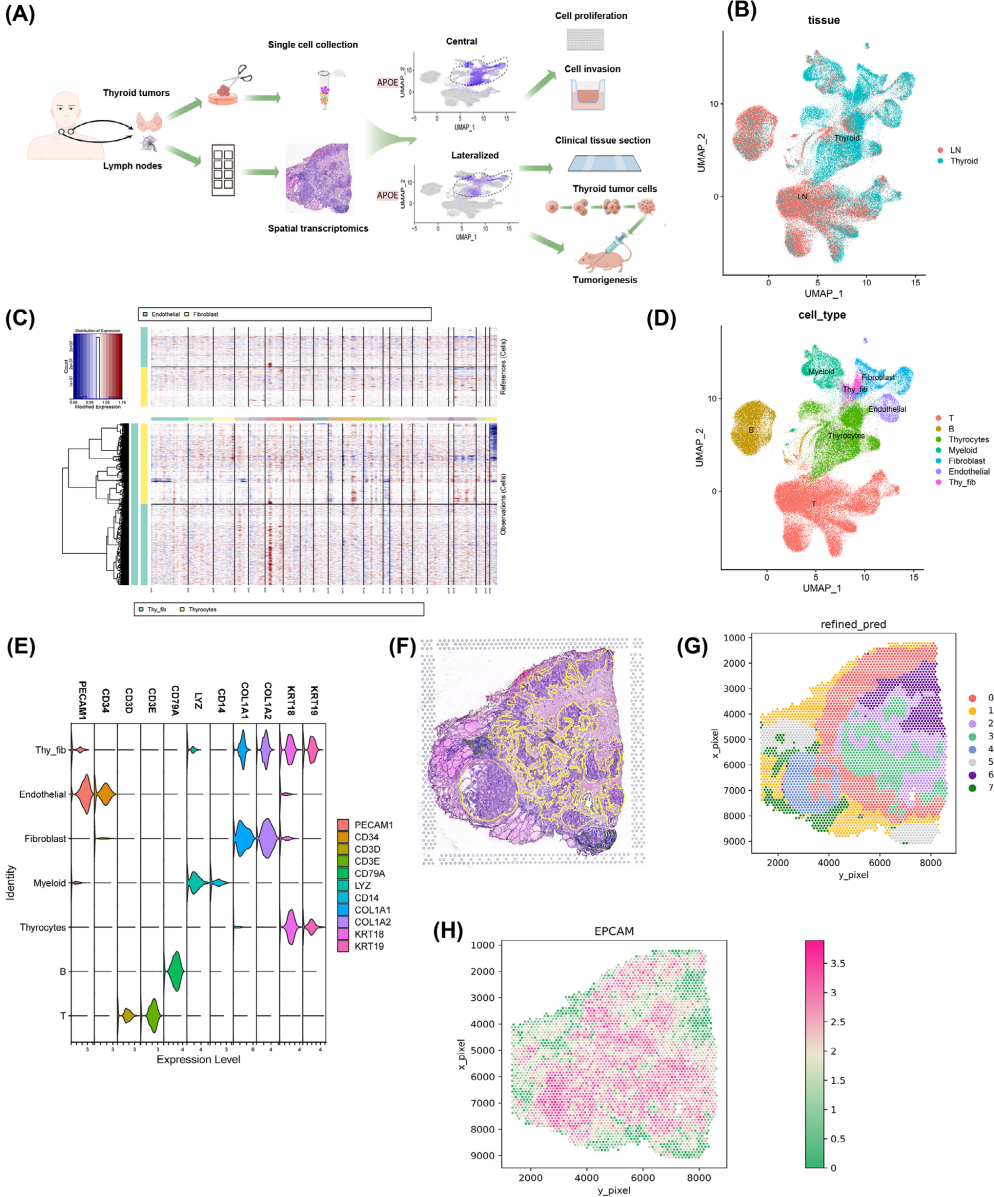
Overview of single transcriptomic and spatial transcriptomic profiling of thyroid cancer. (A) Scheme of the study; Thyroid: thyroid primary tumours. (B) Tissue origin of cells demonstrated in 2D UMAP. The colour of cells represents the origin (blue: Thyroid; red: Lymph node). (C) Inferred cellular ploidy was demonstrated in a 2D UMAP projection. The colour of cells represents the ploidy (blue: diploid; red: aneuploid). (D) Visualization of major cell type annotations in 2D UMAP. (E) Violin plots demonstrated marker genes used to annotate major cell types. (F) Pathology annotation of the example histology slide from patients. Yellow: Tumour region; Green: lymphoid tissue. (G) Domains defined by SpaGCN. Different colours demonstrated different domains. (H) EPCAM expression profile of the example slides. Green to Red, expression level low to high.

Seven patients underwent thyroidectomy for thyroid tumours with a pathologically confirmed diagnosis of PTC. Following surgery, 12 specimens of primary tumour resections (six regional lymph nodes and six thyroid tumours) were obtained and dissociated for single‐cell sequencing. Of these 12 specimens, six (three regional lymph nodes and three thyroid tumours) were sectioned and subjected to spatial transcriptomics (Figure 1A). We detected metastases in the lymph nodes surrounding the thyroid gland, predominantly in the central region, followed by cervical lymph node metastases (LNMs). The number and location of LNMs were significantly correlated with the patient prognosis and the surgical employed for thyroid cancer. Details of the patients and their diagnoses are presented in Table 1.

**TABLE 1:** Samples information, related to Figure 1.

Patient ID	Tissue origins	Gender	Age	Tumour tissue cellularity	Histologi csubtype	TNM stage
P1	P1T: Primary tumour P1L: Metastatic lymph nodes P2T: Primary tumour	Female	43	Papillary thyroid carcinoma	Classica	T1bN1aM0
P2	P2T: Primary tumour P2L: Metastatic lymph nodes	Female	41	Papillary thyroid carcinoma	Classica	T2N1bM0
P3	P3L: Metastatic lymph nodes	Male	44	Papillary thyroid carcinoma	Classica	T2N1bM0
P4	P4T: Primary tumour	Male	23	Papillary thyroid carcinoma	Classica	T1bN1bM0
P5	P5T: Primary tumour P5L: Normal lymph nodes	Female	43	Papillary thyroid carcinoma	Classica	T1bN1bM0
P6	P6T: Primary tumour P6L: Metastatic lymph nodes	Male	53	Papillary thyroid carcinoma	Classica	T2N1bM0
P7	P7T: Primary tumour P7L: Metastatic lymph nodes	Male	53	Papillary thyroid carcinoma	Classica	T2N1bM0
P8	P8T: Primary tumour P8L: Metastatic lymph nodes	Female	59	Medullary thyroid carcinoma	/	T2N1bM0

We combined scRNA‐seq data from all samples, treating each sample as a separate batch, using a batch‐balanced k‐nearest neighbours algorithm. After stringent quality control, 144 746 cells were retained, with a median gene detection count of 1414 per cell. We visualized the cellular landscape using uniform manifold approximation and projection (UMAP), highlighting tissue, sample and patient origins (Figure 1B and Figure S1A,B). Notably, we observed substantial cellular diversity at both the sample and patient levels. To distinguish between tumour and normal cells, we assessed cellular ploidy using CopyKat and InferCNV algorithms on a randomly downsampled subset of the data (Figure 1C and Figure S2). We annotated cell types based on marker gene expression (*CD3D* and *CD3E* for T cells, *CD79A* for B cells, *LYZ* and *CD14* for myeloid cells, *COL1A1* and *COL1A2* for fibroblast cells, *PECAM1* and *CD34* for endothelial cells, and *KRT18* and *KRT19* for thyrocytes) (Figure 1D,E), as described in a previous study.^13^ To isolate the heterogeneity specific to PTC, we generated a separate UMAP plot excluding the medullary thyroid carcinoma (MTC) case, as its tumour origin differs (Figure S3). Interestingly, fibroblasts, thyrocytes and endothelial cells exhibited aneuploidy and a novel cellular state expressing both thyrocyte and fibroblast markers, termed ‘thy_fib.’. At the patient level, heterogeneity was primarily observed in tumour cells, as anticipated from previous studies. To further explore this, we plotted the number and proportions of different cell types (Figure S4) and generated individual UMAP plots (Figure S5).

We performed transcriptomic profiling of the cohort to annotate the collected spatial transcriptomic data. A representative of a histological slide prepared for spatial transcriptomic analysis is depicted in Figure 1F. Employing SpaGCN,^14^ we identified distinct domains (Figure 1G). Notably, we successfully delineated tumour regions exhibiting differential *EPCAM* expression, aligning with the expert pathologist's assessment (Figure 1H and Figure S6).

### 2.2 Lower *APOE* gene expression is associated with tumour cell metastasis

**FIGURE 2:**
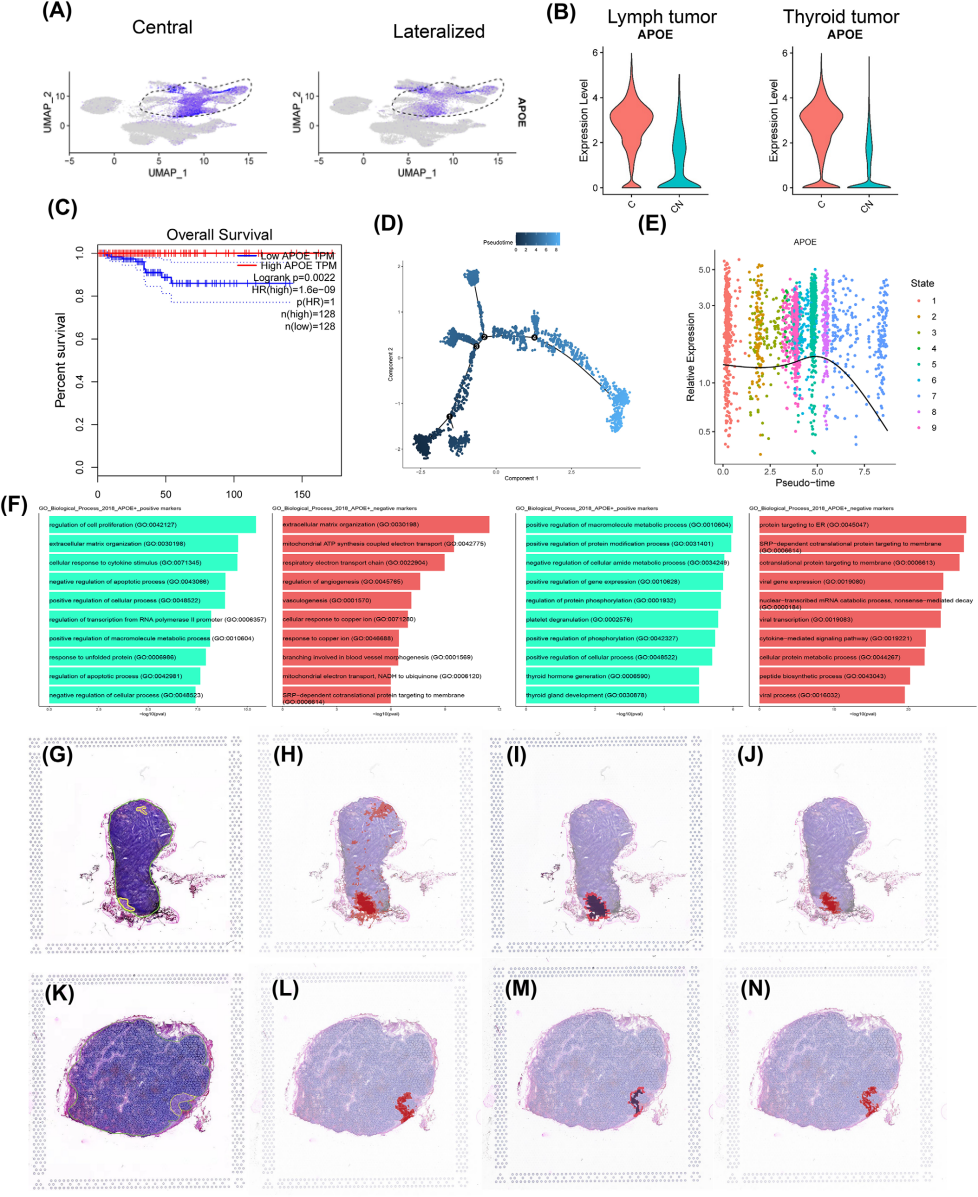
*APOE* gene expression is associated with tumour cell metastasis. (A) Distribution of APOE expression in cells from samples with different metastasis characters (left: centre metastasis; right: centre plus neck metastasis). (B) *APOE* expression in lymph node and thyroid tumour cells from samples with different metastasis characters. Lymph tumour: metastatic tumour cells in LN; Thyroid tumour: Thyroid primary tumours. (C) TCGA survival analysis of PTC patients regarding different *APOE* expression levels. *N* = 256. (D) Pseudo‐time trajectory for tumour cells. The colour of cells represents the inferred pseudo‐time. (E) *APOE* expression along the pseudo‐time. Each data point represents the *APOE* expression in a cell. The colour of cells represents the inferred states of tumour cells along the trajectory. (F) Pathways (GO Biological Process) enriched in differentially expressed gene sets between *APOE* ^+^ and *APOE* ^−^ cells (left two panels for thyroid tumour location; right two panels for lymph node location). (G, K) Pathology annotation of two lymph node histology slides from two patients with metastatic PTC. Yellow: Tumour region. (H, L) Tumour region identified by TESLA using four indicated marker expression levels. (I, M) Tumour Edge detected by TESLA. The red colour indicates the tumour edge, while purple denotes the tumour core. (J−N) APOE expression marked by TESLA DE program, red highlights the region where APOE is significantly overexpressed.

Consistent with these findings, lower *APOE* expression was significantly associated with poorer overall survival in patients with PTC from The Cancer Genome Atlas (TCGA) database (*n* = 256, Figure 2C). To further investigate the role of APOE in tumour progression, we performed sub‐clustering of tumour cells (Figure S8A) and pseudo‐time trajectory analysis using Monocle 3 (Figure 2D,E and Figure S8B). This analysis identified nine distinct cellular states based on their position along the trajectory (Figure S8C). When examining *APOE* expression across these 13 tumour subclusters (Figure S8D), we observed a striking correlation between lower *APOE* levels and later‐stage tumour cell states. This finding suggests that late‐stage tumour cells, characterized by lower *APOE* expression, may contribute more significantly to metastatic progression.

We partitioned tumour cells into two groups based on *APOE* expression, acknowledging that the zero‐inflation inherent to single‐cell data might introduce some degree of error. Our goal was to identify major trends. We identified differentially expressed genes (DEGs) between *APOE* ^+^ and *APOE* ^−^ tumour cells in both thyroid and lymph node samples (Figure S9). Subsequently, we performed pathway enrichment analysis on these gene sets (Figure 2F). In thyroid *APOE* ^−^ cells, enriched pathways were associated with enhanced metabolic processes, such as the respiratory electron transport chain and angiogenesis regulation. In contrast, lymph node *APOE* ^−^ cells exhibited enrichment in pathways related to protein synthesis, including peptide biosynthesis and protein targeting to the endoplasmic reticulum.

We examined *APOE* expression in histological slides of lymph node tissues containing metastatic tumours. Employing a novel algorithm capable of super‐resolution analysis of tumour ecosystems,^15^we first identified tumour cells based on the expression of *EPCAM*, *TG*, *KRT18* and *KRT19*. This automated segmentation closely aligned with the tumour regions delineated by expert pathologists (Figure 2G,H,K,L). Similarly, the main tumour edge was defined using these marker sets (Figure 2I,M). Intriguingly, we observed a higher level of *APOE* expression within the core of the tumour compared to the tumour edge (Figure 2J,N). This finding suggests that *APOE*‐expressing tumour cells may play a crucial role in facilitating lymph node metastasis.

### 2.3 Overexpression of *APOE* inhibits tumour cell proliferation and invasion in vitro

**FIGURE 3:**
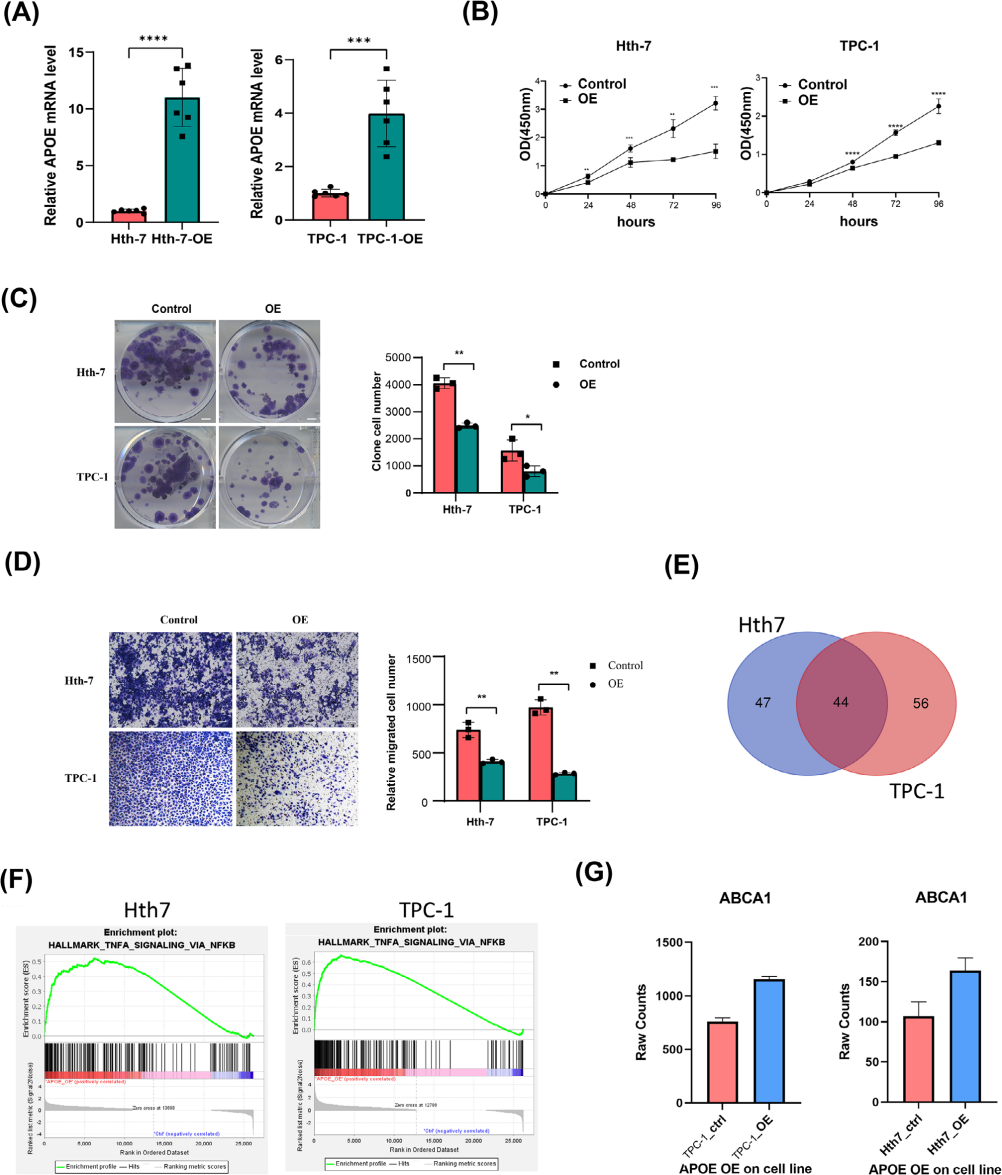
Overexpression of *APOE* inhibits tumour cell proliferation and invasion in vitro. (A) Hth‐7 and TPC‐1 cells were transfected with control and *APOE*‐overexpression lentivirus (OE) for 48 h. Cell lines that stably overexpressed *APOE* were obtained through puromycin selection. Total cellular RNA was extracted for RT‐PCR analysis. Overexpression efficiencies were verified via RT‐PCR. *p < .05; **p < .01; ***p < .001. (B, C) The proliferation of indicated cells was analysed with a Cell Counting Kit‐8 (CCK8) assay (B) and colony formation (C). Control and OE cells were cultured and evaluated at 0, 24, 48, 72 and 96 h. *n* = 5 biologically independent experiments. Colony formation was assessed after 2 weeks. The results from three independent experiments are indicated as means ± standard deviations. (D) Transwell invasion analysis revealed the invasive ability of cells. (E) Each bar represents the mean ± standard deviation of three independent experiments. (F) GSEA enrichment analysis reveals that ‘TNFA_signaling_via_NFKB’ is the most enriched hallmark pathway in both cell lines. (G) The bar plot displays the RNA‐seq raw counts (raw expression levels) of ABCA1 in both cell lines, with and without *APOE* overexpression.

We further validated the role of *APOE* gene expression in PTC using scRNA‐seq data. We established *APOE* overexpression Hth‐7 and TPC‐1 cell lines via lentiviral transfection (Figure 3A). To assess the impact of *APOE* overexpression on cellular behaviour, we performed CCK‐8 assays and colony formation assays. These experiments revealed a significant decrease in the proliferation of *APOE*‐overexpressing cells (Figure 3B,C). Additionally, Matrigel invasion chamber assays suggested a substantial reduction in the invasive ability of these cells (Figure 3D). These results collectively indicate a potential role for *APOE* gene expression in inhibiting PTC progression. To explore the underlying mechanisms, we conducted transcriptome sequencing analysis of *APOE*‐overexpressing Hth‐7 and TPC‐1 cell lines. This analysis identified 44 overlapping DEGs with an FDR‐adjusted *p‐*value < .05, |log2foldchange| > 1 and raw counts > 10 (Figure 3E). Gene set enrichment analysis revealed that genes associated with the ‘Hallmark_TNFa_Signaling_via_NFKB’ pathway were positively correlated with *APOE* overexpression in both cell lines (Figure 3F). This pathway is closely linked to immune responses and tumour progression. Notably, *ABCA1*, a gene previously reported as critical for *APOE* lipidation,^16^ was significantly upregulated in both *APOE‐*overexpressing cell lines (Figure 3G). *ABCA1*, in conjunction with *APOE*, may contribute to the activation of the *LXR* transcription factor, thereby potentially restricting immunosuppression.^17^ These findings suggest a complex interplay between *APOE*, *ABCA1* and *LXR* that may influence tumour immunity and progression.

Furthermore, KEGG pathway enrichment analysis revealed significant enrichment of metabolism‐related and immune‐related pathways in *APOE*‐overexpressing cells (Figure S10A). To gain deeper insights into potential metabolic changes, we performed an untargeted Liquid Chromatograph Mass Spectrometer (LC‐MS) metabolomics analysis. Metabolites were analysed for fold change between the groups, and a *t*‐test with criteria of VIP ≥ 1 and *p* < .05 was conducted to identify significant differences (Figure S10B). A one‐way ANOVA was utilized for statistical comparisons, followed by post‐hoc pairwise comparisons (Figure S10C). The volcano plot visualized the differential metabolites between the two groups (Figure S10D,E). Additionally, KEGG pathway analysis revealed significant enrichment of metabolites (Figure S10F,G). Collectively, these data suggest that *APOE* may influence the tumour microenvironment and progression through multiple pathways.

### 2.4 In vivo validation of *APOE* function in tumour inhibition and large‐scale human validation

**FIGURE 4:**
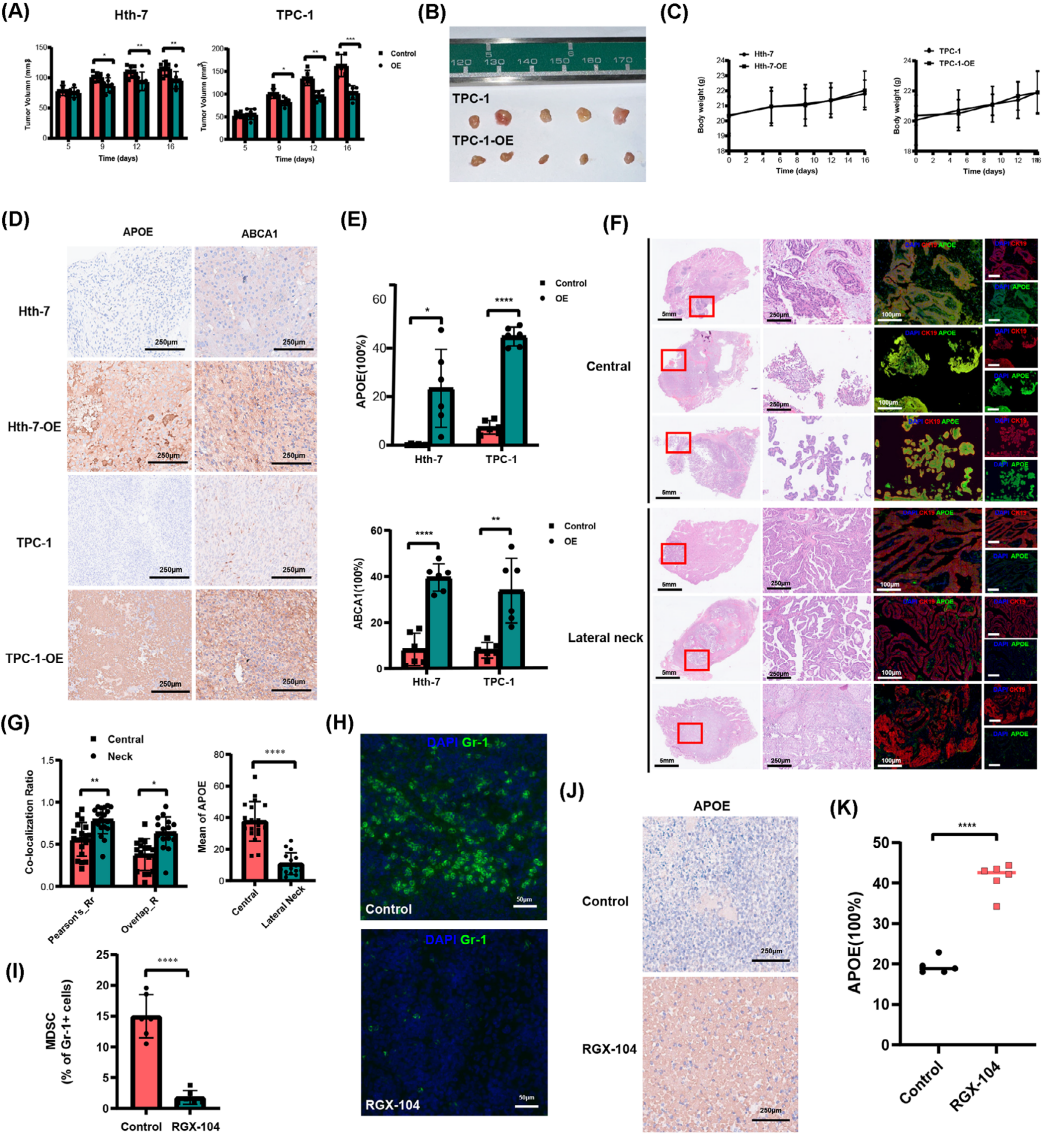
In vivo validation of *APOE* function in tumour inhibition and its large‐scale human validation. (A–C) *APOE* overexpression inhibited xenograft tumours. 1×10^7^ *APOE*‐overexpression Hth‐7 and TPC‐1 cells and control cells were subcutaneously grafted in athymic, female nude mice. Tumour volume (A, B) and body weight (C) were measured every 3−4 days (volume  =  width^2^ × length × 1/2) (*p < .05; **p < .01; ***p < .001). (D) Tumour tissues were removed from mice after 2 weeks and slides were immunohistochemically stained with APOE and *ABCA1*. (E) Quantification results for the *APOE* and *ABCA1* expression of the immunohistochemistry analysis. (F) Tumour sections were haematoxylin and eosin (HE)‐stained and observed via dual colour fluorescence. Fluorescent images of thyroid tumours in which *CK19*‐positive cells are stained red and *APOE*‐positive cells are stained green. Nuclei were stained blue (DAPI). (G) Quantification of the colocalization of *APOE* and *CK19* immunofluorescence data. (H) Representative are images of Gr‐1 immunofluorescence staining in cells treated with saline (top) or RGX‐104 (bottom). Green staining indicates Gr‐1‐positive cells, while blue staining marks nuclei. (I) Quantification results for the Gr‐1‐positive cells of the immunofluorescence analysis. (J) Representative images of *APOE* expression after treatment with the saline (top) or RGX‐104 (bottom). (K) Quantification results for the *APOE* expression of the immunohistochemistry analysis.

Next, we sought to validate these findings in vivo. We utilized *APOE*‐overexpressing cells for subcutaneous xenograft experiments in athymic, female nude mice. Notably, *APOE* overexpression significantly inhibited the tumorigenicity of PTC cells in vivo. The tumour growth of *APOE*‐expressing cells was substantially slower compared to the control group (Figure 4A,B), while these genetic modifications did not affect mouse body weight (Figure 4C). We further performed immunohistochemical analysis to assess *APOE* and *ABCA1* expression in tumour tissues. Our results demonstrated that *APOE* overexpression was accompanied by increased *ABCA1* expression in tumour tissues (Figure 4D,E).

To further validate the clinical relevance of *APOE* expression in metastatic PTC, we investigated *APOE* expression in a cohort of 41 patients with metastatic disease. Immunofluorescence staining revealed a significant decrease in co‐expression of *APOE* and *CK19* in patients with lateral cervical LNM compared to those with central compartment LNMs (Figure 4F,G). This suggests that patients with lateral cervical LNM exhibit lower *APOE* expression levels in tumour cells relative to normal cells. Considering the link between *LXR* and *APOE*, we administered intraperitoneal injections of the *LXR*‐agonist RGX‐104 to mice. Treatment with RGX‐104 resulted in a suppression of myeloid‐derived suppressor cells (MDSCs) and a concomitant increase in *APOE* expression (Figure 4H−K). Collectively, these findings suggest a potential role for *LXR* in regulating *APOE* expression. Moreover, the observed inhibition of tumour growth in vitro and in vivo by *APOE* overexpression supports the hypothesis that *APOE* may play a tumour‐suppressive role.

### 2.5 Ligand‐receptor pairs driving differential immune−tumour interactions associated with *APOE* expression profiles

**FIGURE 5:**
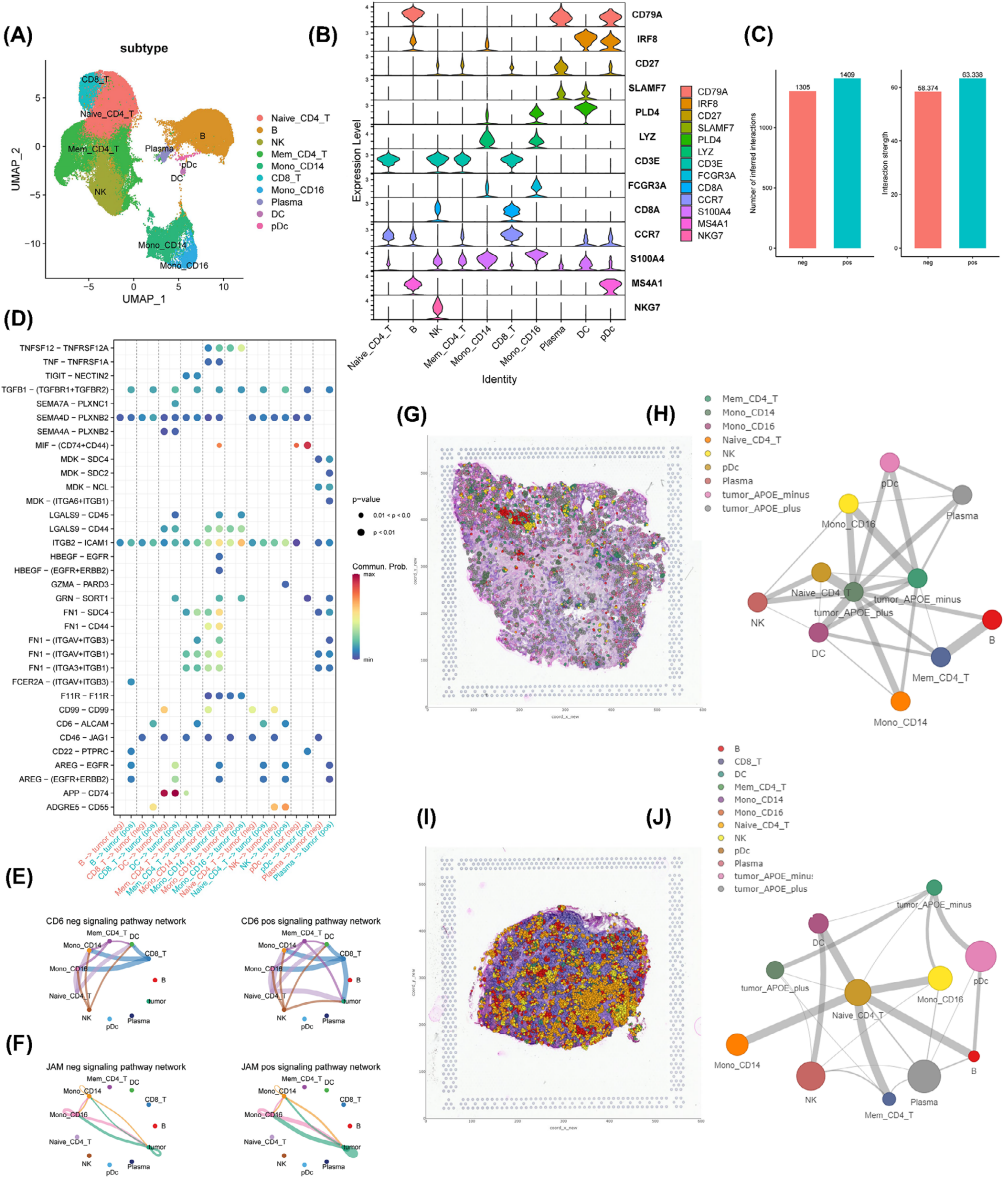
Differential immune−tumour interactions associated with APOE expression profiles. (A) Visualization of re‐clustered immune cell type annotation in 2D UMAP. (B) Violin plots demonstrating marker genes used to annotate immune cell types. (C) Summary of overall interaction between immune cells with *APOE^+^* tumour cells and immune cells with *APOE* ^−^ tumour cells (left total numbers of interaction events; right: normalized interaction strength). The figure illustrates the total number of interactions and interaction strength within the inferred cell−cell communication networks between the ‘APOE neg’ and ‘APOE pos’ groups, as determined using CellChat. (D) Overview of ligand‐receptor pairs showing differential interaction profiles between the *APOE^+^* and *APOE* ^−^ cells (with immune cells). (E, F) Demonstration of signalling pathway network of CD6 (E) and JAM (F). The width of the strings represents the interaction strength of the interaction. Neg: tumour cells were *APOE*‐negative cells; POS: tumour cells were *APOE*‐positive cells; the figure showcases a representative differentially expressed ligand‐receptor pathway, *CD6*, obtained from the CellChat package. This pathway demonstrates the variations in cell communication between the ‘*APOE* neg’ and ‘*APOE* pos’ groups. (G, I) CellTrek analysis mapped the specific cell type back to the histology slides from different tissues (G: primary tumour, I: Lymph node). The figure represents the co‐embedding analysis of spatial transcriptomic (ST) and single‐cell RNA‐seq (scRNA‐seq) datasets utilizing the CellTrek ‘traint’ function. The objective of this step is to assess the overlap between these two data modalities. Following the co‐embedding process, single cells can be mapped to their spatial positions. (H, J) Cell−cell colocalization/interaction analysis of different cell types inferred by CellTrek analysis.

We sought to leverage the comprehensive profiling of immune cells in our cohort by performing a sub‐clustering of these cells (Figure 5A). We used established markers to annotate immune cells into 10 distinct types (Figure 5B): naïve CD4^+^ T cells, memory CD4^+^ T cells, CD8^+^ T cells, natural killer (NK) cells, B cells, plasma cells, plasmacytoid dendritic cells, dendritic cells (DCs), CD14^+^ monocytes and CD16^+^ monocytes. A comparative analysis of samples with different metastatic conditions, specifically central, central plus neck (CN) and healthy (none), revealed dramatic shifts in the abundance of certain immune cell types. Notably, the CN group exhibited a clear enrichment of both CD14^+^ and CD16^+^ monocytes, coupled with a depletion of CD8^+^ T cells (Figure S11).

We further investigated the interaction between *APOE* ^+^/*APOE* ^−^ tumour cells with immune cells in lymph nodes. We found that *APOE* ^−^ cells stimulated the immune system to a lesser degree than *APOE* ^+^ cells, regardless of the normalization method employed (Figure 5C). We identified several ligand‐receptor pairs that underlie this pattern (Figure 5D), including the CD6‐ and F11R‐driven signalling pathway networks. Specifically, *APOE* ^+^ tumour cells were involved in the CD6‐driven network, along with immune cells (Figure 5E). For F11R (JAM1), both *APOE* ^+^ and *APOE* ^−^ cells interacted with immune cells (Figure 5F), with *APOE* ^+^ cells exhibiting stronger interactions. Interestingly, in contrast to *APOE* ^+^ cells, *APOE* ^−^ cells specifically interacted with immune cells, such as DCs, through a CD99‐driven network rather than the CD6‐driven network. To further explore the role of the CD99‐driven network in the interaction between *APOE*‐positive and APOE‐negative tumour cells and immune cells, we focused on these cell types at both the primary thyroid site and lymph node metastasis site (Figure S12). Notably, in the *APOE*‐negative group, the ESAM and CD99 pathways exhibited strong interactions at both sites. Furthermore, the CD46 pathway, which is linked to immunosuppression,^18^, ^19^ showed stronger interactions between *APOE*‐negative tumour cells and immune cells, particularly at the lymph node metastasis site. Using CellTrek,^20^ we directly visualized the communication networks between *APOE* ^−^ and *APOE* ^+^ tumour cells and immune cells on histological slides. Strikingly, *APOE* ^−^ tumour cells exhibited minimal interaction with NK, B and T cells in both primary tumour tissues (Figure 5G,H) and lymph nodes (Figure 5I,J), while *APOE* ^+^ tumour cells strongly interacted with these cell types.

To further confirm the association of *APOE* with a suppressive immune microenvironment, we generated APOE knockout mice using a CRISPR‐Cas9 system and verified the knockout by PCR (Figure S13A,B). We observed a decrease in mature DCs, NK cells and NKT cells in *APOE* ^−^/^−^ mice (Figure S13C,D). Additionally, the M1/M2 macrophage ratio was also reduced (Figure S13E). However, the numbers of CD4^+^ and CD8^+^ T cells did not differ significantly (Figure S13F). These spatial data suggest that *APOE*‐negative tumour cells may promote tumour growth and invasion by creating an immunosuppressive microenvironment.

### 2.6 Machine learning‐based model predicts metastasis patterns

**FIGURE 6:**
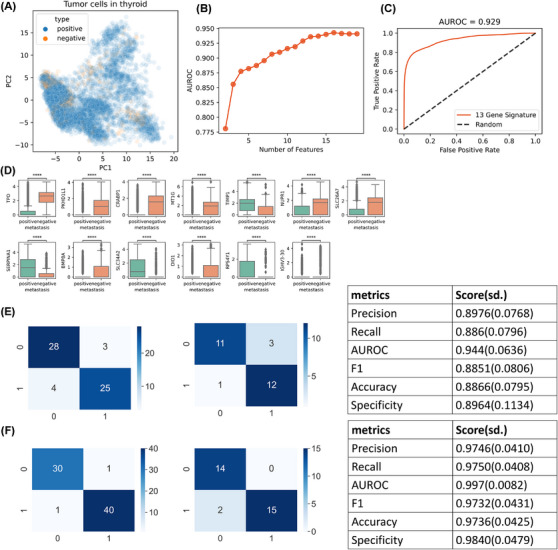
Prediction of PTC metastasis and its location by machine learning‐based model. (A) 2D embeddings (principal components 1 and 2) of thyroid tumour cells. Cell origins were labelled with different colours depending on the patient's phenotype (positive: with metastasis; negative: no metastasis). (B) AUROC scores for random forest models using different numbers of genes as input. (C) AUROC curve for final random forest model using 13 genes as input. (D) Expression levels of final 13‐gene signature of thyroid tumour cells. (E) Confusion matrix for the training set (left) and test set (middle) of the prediction model with qPCR data from surgical samples, and the model performance (table right). (F) Confusion matrix for the training set (left) and test set (middle) of the prediction model with qPCR data from biopsy samples, and the model performance (table right).

Given the significant morbidity and mortality associated with metastatic thyroid cancer, early prediction of metastatic potential is crucial for improved patient outcomes. We leveraged our comprehensive single‐cell atlas to develop a predictive model capable of effectively classifying patients into high‐ and low‐risk groups for metastasis. We used only tumour cells to build a predictive model to distinguish between the two groups (Figure 6A). These cells did not clearly differ in their patterns of metastasis in the latent space constructed by principal component analysis (PCA). While incorporating all genes into the model was theoretically possible, we aimed to simplify the model and facilitate the development of practical diagnostic assays by limiting the number of genes. To achieve this, we ranked genes based on their mutual information scores. We constructed random forest classifiers using varying numbers of top‐ranked genes and evaluated their performance. The model's performance plateaued after incorporating the top 13 genes with the highest mutual information scores (Figure 6B). This 13‐gene model demonstrated excellent predictive power, with an area under the receiver operating characteristic curve (AUROC) of .929 (Figure 6C). We visualized the expression patterns of these 13 genes in tumour cells from the high‐ and low‐risk metastasis groups (Figure 6D), revealing distinct expression profiles between the two groups. Notably, nine of these 13 genes were upregulated in the *APOE*‐negative group, suggesting that their association with metastasis is not directly linked to *APOE* metabolism. To validate the model's clinical utility, we collected an additional 203 clinical samples obtained through surgery or biopsy and performed qPCR for the 13‐gene panel. Recognizing the potential differences between qPCR and scRNA‐seq data, we retrained the model separately for surgical and biopsy samples, using 70% of the data for training and the remaining 30% for validation. Both models exhibited excellent performance, with high scores for precision, recall and AUROC (Figure 6E,F). Detailed patient information and diagnostic outcomes are presented in Table 3.

In addition to the presence or absence of metastasis, the pattern of metastasis (central or CN) is also clinically significant. To address this, we employed a similar machine‐learning approach to distinguish between these two conditions. Consistently, only tumour cells were utilized to build the model (Figure S13A). We initially developed a model based on single‐cell data, incorporating 10 genes as features (Figure S13B−D). To further validate and refine the model, we collected additional clinical samples through biopsy. Unfortunately, we did not obtain a sufficient number of surgical samples for this purpose. Using qPCR data from the biopsy samples, we retrained the model and achieved satisfactory performance (Figure S13E). We are currently developing formal, rapid‐diagnostic approaches based on these trained models.

## 3 MATERIALS AND METHODS

### 3.1 Human participants

A total of eight thyroid cancer patients were enrolled in this study. Fourteen samples were selected for single‐cell transcriptome sequencing, and eight samples were included for spatial sequencing. Among these patients, seven had papillary thyroid carcinoma, and one had medullary thyroid carcinoma. Detailed clinical information for these patients is provided in Table 1. Tissue sections from 41 PTC surgical specimens were selected for immunofluorescence staining. Clinical information for these patients is available in Table 2. A total of 203 thyroid cancer tissue DNA samples were obtained from punch biopsies of patients undergoing thyroidectomy. Clinical information for these patients is described in Table 3. All human tissue samples used in this study were obtained with approval from the Human Ethics Committee of Ruijin Hospital, Shanghai Jiao Tong University School of Medicine, Shanghai, China (2023LLSDN11).

**TABLE 2:** The clinical specimen information of the patients for immunofluorescent colocalization.

Patient ID	Gender	Age	Tumour tissue cellularity	Type of tissue	Lymph node metastases	TNM stage
P9‐T	Female	17	Papillary thyroid carcinoma	Primary lesion	Lateralized	T3aN1bM0
P10‐T	Female	23	Papillary thyroid carcinoma	Primary lesion	Lateralized	T1bN1bM0
P11‐T	Female	25	Papillary thyroid carcinoma	Primary lesion	Lateralized	T1aN1bM0
P12‐T	Female	29	Papillary thyroid carcinoma	Primary lesion	Lateralized	T2N1bM0
P13‐T	Male	30	Papillary thyroid carcinoma	Primary lesion	Lateralized	T1bN1bM0
P14‐T	Male	32	Papillary thyroid carcinoma	Primary lesion	Lateralized	T2N1bM0
P15‐T	Female	33	Papillary thyroid carcinoma	Primary lesion	Lateralized	T2N1bM0
P16‐T	Female	33	Papillary thyroid carcinoma	Primary lesion	Lateralized	T1aN1bM0
P17‐T	Male	36	Papillary thyroid carcinoma	Primary lesion	Lateralized	T3aN1bM0
P18‐T	Male	37	Papillary thyroid carcinoma	Primary lesion	Lateralized	T2N1bM0
P19‐T	Female	39	Papillary thyroid carcinoma	Primary lesion	Lateralized	T2N1bM0
P20‐T	Male	46	Papillary thyroid carcinoma	Primary lesion	Lateralized	T1bN1bM0
P21‐T	Female	48	Papillary thyroid carcinoma	Primary lesion	Lateralized	T1bN1bM0
P22‐T	Female	49	Papillary thyroid carcinoma	Primary lesion	Lateralized	T2N1bM0
P23‐T	Male	50	Papillary thyroid carcinoma	Primary lesion	Lateralized	T1aN1bM0
P24‐T	Male	50	Papillary thyroid carcinoma	Primary lesion	Lateralized	T2N1bM0
P25‐T	Male	51	Papillary thyroid carcinoma	Primary lesion	Lateralized	T3aN1bM0
P26‐T	Female	59	Papillary thyroid carcinoma	Primary lesion	Lateralized	T2N1bM0
P27‐T	Male	65	Papillary thyroid carcinoma	Primary lesion	Lateralized	T1bN1bM0
P28‐T	Female	21	Papillary thyroid carcinoma	Primary lesion	Central	T2N1bM0
P29‐T	Male	22	Papillary thyroid carcinoma	Primary lesion	Central	T2N1bM0
P30‐T	Female	23	Papillary thyroid carcinoma	Primary lesion	Central	T2N1bM0
P31‐T	Male	26	Papillary thyroid carcinoma	Primary lesion	Central	T1bN1bM0
P32‐T	Female	29	Papillary thyroid carcinoma	Primary lesion	Central	T1bN1bM0
P33‐T	Male	30	Papillary thyroid carcinoma	Primary lesion	Central	T1bN1bM0
P34‐T	Female	31	Papillary thyroid carcinoma	Primary lesion	Central	T1bN1bM0
P35‐T	Female	33	Papillary thyroid carcinoma	Primary lesion	Central	T2N1bM0
P36‐T	Female	37	Papillary thyroid carcinoma	Primary lesion	Central	T1bN1bM0
P37‐T	Male	39	Papillary thyroid carcinoma	Primary lesion	Central	T1aN1bM0
P38‐T	Female	39	Papillary thyroid carcinoma	Primary lesion	Central	T1aN1bM0
P39‐T	Male	46	Papillary thyroid carcinoma	Primary lesion	Central	T1bN1bM0
P40‐T	Female	46	Papillary thyroid carcinoma	Primary lesion	Central	T1bN1bM0
P41‐T	Male	46	Papillary thyroid carcinoma	Primary lesion	Central	T1bN1bM0
P42‐T	Female	48	Papillary thyroid carcinoma	Primary lesion	Central	T2N1bM0
P43‐T	Male	50	Papillary thyroid carcinoma	Primary lesion	Central	T1aN1bM0
P44‐T	Female	51	Papillary thyroid carcinoma	Primary lesion	Central	T2N1bM0
P45‐T	Female	57	Papillary thyroid carcinoma	Primary lesion	Central	T3aN1bM0
P46‐T	Female	59	Papillary thyroid carcinoma	Primary lesion	Central	T1bN1bM0
P47‐T	Male	59	Papillary thyroid carcinoma	Primary lesion	Central	T1bN1bM0
P48‐T	Female	64	Papillary thyroid carcinoma	Primary lesion	Central	T2N1bM0
P49‐T	Female	68	Papillary thyroid carcinoma	Primary lesion	Central	T1aN1bM0

**TABLE 3:** The clinical specimen information of the patients for marker validation.

Patient ID	Gender	Age	Tumour tissue cellularity	Type of tissue	Lymph node metastases	TNM stage
P50‐T	Female	66	Papillary thyroid carcinoma	Primary lesion	None	T1aN0M0
P51‐T	Female	46	Papillary thyroid carcinoma	Primary lesion	None	T1aN0M0
P52‐T	Female	46	Papillary thyroid carcinoma	Primary lesion	None	T1aN0M0
P53‐T	Male	56	Papillary thyroid carcinoma	Primary lesion	None	T1aN0M0
P54‐T	Female	48	Papillary thyroid carcinoma	Primary lesion	None	T1aN0M0
P55‐T	Female	48	Papillary thyroid carcinoma	Primary lesion	None	T1aN0M0
P56‐T	Female	53	Papillary thyroid carcinoma	Primary lesion	None	T1aN0M0
P57‐T	Female	51	Papillary thyroid carcinoma	Primary lesion	None	T1aN0M0
P58‐T	Male	27	Papillary thyroid carcinoma	Primary lesion	None	T1aN0M0
P59‐T	Female	49	Papillary thyroid carcinoma	Primary lesion	None	T1aN0M0
P60‐T	Female	35	Papillary thyroid carcinoma	Primary lesion	None	T1aN0M0
P61‐T	Male	37	Papillary thyroid carcinoma	Primary lesion	None	T1aN0M0
P62‐T	Female	25	Papillary thyroid carcinoma	Primary lesion	None	T1aN0M0
P63‐T	Female	37	Papillary thyroid carcinoma	Primary lesion	None	T1aN0M0
P64‐T	Male	32	Papillary thyroid carcinoma	Primary lesion	None	T1aN0M0
P65‐T	Female	55	Papillary thyroid carcinoma	Primary lesion	None	T1aN0M0
P66‐T	Male	30	Papillary thyroid carcinoma	Primary lesion	None	T1aN0M0
P67‐T	Female	28	Papillary thyroid carcinoma	Primary lesion	None	T1aN0M0
P68‐T	Female	37	Papillary thyroid carcinoma	Primary lesion	None	T1aN0M0
P69‐T	Female	42	Papillary thyroid carcinoma	Primary lesion	None	T1aN0M0
P70‐T	Female	48	Papillary thyroid carcinoma	Primary lesion	None	T1aN0M0
P71‐T	Female	30	Papillary thyroid carcinoma	Primary lesion	None	T1aN0M0
P72‐T	Female	53	Papillary thyroid carcinoma	Primary lesion	None	T1aN0M0
P73‐T	Female	55	Papillary thyroid carcinoma	Primary lesion	None	T1aN0M0
P74‐T	Male	62	Papillary thyroid carcinoma	Primary lesion	None	T1aN0M0
P75‐T	Female	48	Papillary thyroid carcinoma	Primary lesion	None	T1aN0M0
P76‐T	Male	27	Papillary thyroid carcinoma	Primary lesion	None	T1aN0M0
P77‐T	Female	41	Papillary thyroid carcinoma	Primary lesion	None	T1aN0M0
P78‐T	Female	41	Papillary thyroid carcinoma	Primary lesion	None	T1aN0M0
P79‐T	Female	51	Papillary thyroid carcinoma	Primary lesion	None	T1aN0M0
P80‐T	Female	47	Papillary thyroid carcinoma	Primary lesion	None	T1aN0M0
P81‐T	Female	25	Papillary thyroid carcinoma	Primary lesion	None	T1aN0M0
P82‐T	Female	50	Papillary thyroid carcinoma	Primary lesion	None	T1aN0M0
P83‐T	Female	60	Papillary thyroid carcinoma	Primary lesion	None	T1aN0M0
P84‐T	Male	40	Papillary thyroid carcinoma	Primary lesion	None	T1aN0M0
P85‐T	Female	35	Papillary thyroid carcinoma	Primary lesion	None	T1aN0M0
P86‐T	Female	66	Papillary thyroid carcinoma	Primary lesion	None	T1aN0M0
P87‐T	Female	41	Papillary thyroid carcinoma	Primary lesion	None	T1aN0M0
P88‐T	Female	31	Papillary thyroid carcinoma	Primary lesion	None	T1aN0M0
P89‐T	Female	65	Papillary thyroid carcinoma	Primary lesion	None	T1bN0M0
P90‐T	Female	65	Papillary thyroid carcinoma	Primary lesion	None	T1aN0M0
P91‐T	Female	59	Papillary thyroid carcinoma	Primary lesion	None	T1aN0M0
P92‐T	Female	50	Papillary thyroid carcinoma	Primary lesion	None	T1aN0M0
P93‐T	Female	34	Papillary thyroid carcinoma	Primary lesion	None	T1aN0M0
P94‐T	Male	36	Papillary thyroid carcinoma	Primary lesion	None	T1aN0M0
P95‐T	Female	55	Papillary thyroid carcinoma	Primary lesion	None	T1aN0M0
P96‐T	Female	33	Papillary thyroid carcinoma	Primary lesion	None	T1aN0M0
P97‐T	Female	33	Papillary thyroid carcinoma	Primary lesion	None	T1aN0M0
P98‐T	Female	25	Papillary thyroid carcinoma	Primary lesion	None	T1aN0M0
P99‐T	Female	38	Papillary thyroid carcinoma	Primary lesion	None	T1aN0M0
P100‐T	Female	54	Papillary thyroid carcinoma	Primary lesion	None	T1aN0M0
P101‐T	Female	55	Papillary thyroid carcinoma	Primary lesion	None	T1aN0M0
P102‐T	Female	58	Papillary thyroid carcinoma	Primary lesion	None	T1aN0M0
P103‐T	Female	31	Papillary thyroid carcinoma	Primary lesion	None	T1aN0M0
P104‐T	Female	26	Papillary thyroid carcinoma	Primary lesion	None	T1aN0M0
P105‐T	Male	49	Papillary thyroid carcinoma	Primary lesion	None	T1aN0M0
P106‐T	Female	55	Papillary thyroid carcinoma	Primary lesion	None	T1aN0M0
P107‐T	Female	25	Papillary thyroid carcinoma	Primary lesion	None	T1aN0M0
P108‐T	Male	79	Papillary thyroid carcinoma	Primary lesion	None	T1aN0M0
P109‐T	Male	53	Papillary thyroid carcinoma	Primary lesion	None	T1bN0M0
P110‐T	Female	25	Papillary thyroid carcinoma	Primary lesion	None	T1aN0M0
P111‐T	Female	49	Papillary thyroid carcinoma	Primary lesion	None	T1bN0M0
P112‐T	Female	49	Papillary thyroid carcinoma	Primary lesion	None	T1bN0M0
P113‐T	Male	36	Papillary thyroid carcinoma	Primary lesion	None	T1aN0M0
P114‐T	Female	41	Papillary thyroid carcinoma	Primary lesion	None	T1aN0M0
P115‐T	Female	44	Papillary thyroid carcinoma	Primary lesion	None	T1bN0M0
P116‐T	Female	44	Papillary thyroid carcinoma	Primary lesion	None	T1aN0M0
P117‐T	Female	72	Papillary thyroid carcinoma	Primary lesion	None	T1aN0M0
P118‐T	Female	41	Papillary thyroid carcinoma	Primary lesion	None	T1aN0M0
P119‐T	Female	27	Papillary thyroid carcinoma	Primary lesion	None	T1aN0M0
P120‐T	Female	54	Papillary thyroid carcinoma	Primary lesion	None	T1aN0M0
P121‐T	Male	32	Papillary thyroid carcinoma	Primary lesion	None	T1aN0M0
P122‐T	Female	51	Papillary thyroid carcinoma	Primary lesion	None	T1aN0M0
P123‐T	Female	28	Papillary thyroid carcinoma	Primary lesion	None	T1aN0M0
P124‐T	Male	45	Papillary thyroid carcinoma	Primary lesion	None	T1aN0M0
P125‐T	Male	30	Papillary thyroid carcinoma	Primary lesion	None	T1aN0M0
P126‐T	Female	44	Papillary thyroid carcinoma	Primary lesion	None	T1aN0M0
P127‐T	Female	34	Papillary thyroid carcinoma	Primary lesion	None	T1aN0M0
P128‐T	Female	45	Papillary thyroid carcinoma	Primary lesion	None	T1aN0M0
P129‐T	Male	41	Papillary thyroid carcinoma	Primary lesion	None	T1bN0M0
P130‐T	Female	38	Papillary thyroid carcinoma	Primary lesion	None	T1aN0M0
P131‐T	Female	50	Papillary thyroid carcinoma	Primary lesion	None	T1aN0M0
P132‐T	Female	34	Papillary thyroid carcinoma	Primary lesion	None	T1aN0M0
P133‐T	Female	62	Papillary thyroid carcinoma	Primary lesion	None	T1aN0M0
P134‐T	Female	51	Papillary thyroid carcinoma	Primary lesion	None	T1aN0M0
P135‐T	Male	31	Papillary thyroid carcinoma	Primary lesion	None	T1bN0M0
P136‐T	Male	31	Papillary thyroid carcinoma	Primary lesion	None	T1bN0M0
P137‐T	Male	52	Papillary thyroid carcinoma	Primary lesion	None	T1aN0M0
P138‐T	Male	31	Papillary thyroid carcinoma	Primary lesion	None	T1bN0M0
P139‐T	Female	59	Papillary thyroid carcinoma	Primary lesion	None	T1aN0M0
P140‐T	Female	48	Papillary thyroid carcinoma	Primary lesion	None	T1aN0M0
P141‐T	Female	33	Papillary thyroid carcinoma	Primary lesion	None	T1aN0M0
P142‐T	Female	53	Papillary thyroid carcinoma	Primary lesion	None	T1bN0M0
P143‐T	Female	21	Papillary thyroid carcinoma	Primary lesion	None	T1bN0M0
P144‐T	Female	57	Papillary thyroid carcinoma	Primary lesion	None	T1aN0M0
P145‐T	Female	69	Papillary thyroid carcinoma	Primary lesion	None	T1aN0M0
P146‐T	Female	19	Papillary thyroid carcinoma	Primary lesion	None	T1bN0M0
P147‐T	Female	40	Papillary thyroid carcinoma	Primary lesion	None	T1aN0M0
P148‐T	Male	51	Papillary thyroid carcinoma	Primary lesion	None	T1aN0M0
P149‐T	Male	64	Papillary thyroid carcinoma	Primary lesion	None	T1aN0M0
P150‐T	Female	50	Papillary thyroid carcinoma	Primary lesion	None	T1aN0M0
P151‐T	Female	32	Papillary thyroid carcinoma	Primary lesion	None	T1bN0M0
P152‐T	Female	31	Papillary thyroid carcinoma	Primary lesion	None	T1aN0M0
P153‐T	Female	50	Papillary thyroid carcinoma	Primary lesion	None	T1aN0M0
P154‐T	Female	40	Papillary thyroid carcinoma	Primary lesion	None	T1aN0M0
P155‐T	Female	34	Papillary thyroid carcinoma	Primary lesion	Central	T1aN1aM0
P156‐T	Female	36	Papillary thyroid carcinoma	Primary lesion	Central	T1aN1aM0
P157‐T	Female	38	Papillary thyroid carcinoma	Primary lesion	Central	T1aN1aM0
P158‐T	Female	33	Papillary thyroid carcinoma	Primary lesion	Central	T1bN1aM0
P159‐T	Female	37	Papillary thyroid carcinoma	Primary lesion	Central	T1bN1aM0
P160‐T	Female	37	Papillary thyroid carcinoma	Primary lesion	Central	T1bN1aM0
P161‐T	Female	28	Papillary thyroid carcinoma	Primary lesion	Central	T1bN1aM0
P162‐T	Female	20	Papillary thyroid carcinoma	Primary lesion	Central	T1bN1aM0
P163‐T	Female	25	Papillary thyroid carcinoma	Primary lesion	Central	T1bN1aM0
P164‐T	Female	30	Papillary thyroid carcinoma	Primary lesion	Central	T1bN1aM0
P165‐T	Female	37	Papillary thyroid carcinoma	Primary lesion	Central	T1bN1aM0
P166‐T	Female	34	Papillary thyroid carcinoma	Primary lesion	Central	T1aN1aM0
P167‐T	Female	35	Papillary thyroid carcinoma	Primary lesion	Central	T1aN1aM0
P168‐T	Female	37	Papillary thyroid carcinoma	Primary lesion	Central	T1aN1aM0
P169‐T	Male	35	Papillary thyroid carcinoma	Primary lesion	Central	T1bN1aM0
P170‐T	Male	35	Papillary thyroid carcinoma	Primary lesion	Central	T1aN1aM0
P171‐T	Male	37	Papillary thyroid carcinoma	Primary lesion	Central	T1aN1aM0
P172‐T	Male	31	Papillary thyroid carcinoma	Primary lesion	Central	T1aN1aM0
P173‐T	Male	36	Papillary thyroid carcinoma	Primary lesion	Central	T1aN1aM0
P174‐T	Female	32	Papillary thyroid carcinoma	Primary lesion	Central	T1aN1aM0
P175‐T	Male	67	Papillary thyroid carcinoma	Primary lesion	Central	T1aN1aM0
P176‐T	Female	25	Papillary thyroid carcinoma	Primary lesion	Central	T1bN1aM0
P177‐T	Female	29	Papillary thyroid carcinoma	Primary lesion	Central	T1bN1aM0
P178‐T	Female	25	Papillary thyroid carcinoma	Primary lesion	Central	T1aN1aM0
P179‐T	Female	61	Papillary thyroid carcinoma	Primary lesion	Central	T1aN1aM0
P180‐T	Female	46	Papillary thyroid carcinoma	Primary lesion	Central	T1aN1aM0
P181‐T	Female	27	Papillary thyroid carcinoma	Primary lesion	Central	T1aN1aM0
P182‐T	Female	44	Papillary thyroid carcinoma	Primary lesion	Central	T1aN1aM0
P183‐T	Female	67	Papillary thyroid carcinoma	Primary lesion	Central	T1aN1aM0
P184‐T	Female	53	Papillary thyroid carcinoma	Primary lesion	Central	T1aN1aM0
P185‐T	Male	63	Papillary thyroid carcinoma	Primary lesion	Central	T1bN1aM0
P186‐T	Male	30	Papillary thyroid carcinoma	Primary lesion	Central	T1aN1aM0
P187‐T	Female	30	Papillary thyroid carcinoma	Primary lesion	Central	T1bN1aM0
P188‐T	Male	45	Papillary thyroid carcinoma	Primary lesion	Central	T1aN1aM0
P189‐T	Female	55	Papillary thyroid carcinoma	Primary lesion	Central	T1bN1aM0
P190‐T	Female	55	Papillary thyroid carcinoma	Primary lesion	Central	T1bN1aM0
P191‐T	Female	28	Papillary thyroid carcinoma	Primary lesion	Central	T1aN1aM0
P192‐T	Female	55	Papillary thyroid carcinoma	Primary lesion	Central	T1bN1aM0
P193‐T	Female	55	Papillary thyroid carcinoma	Primary lesion	Central	T1bN1aM0
P194‐T	Female	32	Papillary thyroid carcinoma	Primary lesion	Central	T1aN1aM0
P195‐T	Female	41	Papillary thyroid carcinoma	Primary lesion	Central	T1aN1aM0
P196‐T	Female	30	Papillary thyroid carcinoma	Primary lesion	Central	T1aN1aM0
P197‐T	Male	58	Papillary thyroid carcinoma	Primary lesion	Central	T1bN1aM0
P198‐T	Female	44	Papillary thyroid carcinoma	Primary lesion	Central	T1aN1aM0
P199‐T	Female	57	Papillary thyroid carcinoma	Primary lesion	Central	T1aN1aM0
P200‐T	Female	40	Papillary thyroid carcinoma	Primary lesion	Central	T1aN1aM0
P201‐T	Female	40	Papillary thyroid carcinoma	Primary lesion	Central	T1aN1aM0
P202‐T	Female	37	Papillary thyroid carcinoma	Primary lesion	Central	T1aN1aM0
P203‐T	Female	24	Papillary thyroid carcinoma	Primary lesion	Central	T21N1aM0
P204‐T	Male	32	Papillary thyroid carcinoma	Primary lesion	Central	T1aN1aM0
P205‐T	Female	43	Papillary thyroid carcinoma	Primary lesion	Central	T1aN1aM0
P206‐T	Male	57	Papillary thyroid carcinoma	Primary lesion	Central	T1aN1aM0
P207‐T	Female	35	Papillary thyroid carcinoma	Primary lesion	Central	T1aN1aM0
P208‐T	Male	46	Papillary thyroid carcinoma	Primary lesion	Central	T1aN1aM0
P209‐T	Male	42	Papillary thyroid carcinoma	Primary lesion	Central	T1bN1aM0
P210‐T	Female	35	Papillary thyroid carcinoma	Primary lesion	Central	T1aN1aM0
P211‐T	Male	39	Papillary thyroid carcinoma	Primary lesion	Central	T1aN1aM0
P212‐T	Female	27	Papillary thyroid carcinoma	Primary lesion	Central	T1aN1aM0
P213‐T	Female	55	Papillary thyroid carcinoma	Primary lesion	Central	T1aN1aM0
P214‐T	Female	50	Papillary thyroid carcinoma	Primary lesion	Central	T1aN1aM0
P215‐T	Female	33	Papillary thyroid carcinoma	Primary lesion	Central	T1bN1aM0
P216‐T	Male	52	Papillary thyroid carcinoma	Primary lesion	Central	T1bN1aM0
P217‐T	Male	33	Papillary thyroid carcinoma	Primary lesion	Central	T1aN1aM0
P218‐T	Female	56	Papillary thyroid carcinoma	Primary lesion	Central	T1bN1aM0
P219‐T	Female	49	Papillary thyroid carcinoma	Primary lesion	Central	T1aN1aM0
P220‐T	Female	49	Papillary thyroid carcinoma	Primary lesion	Central	T1bN1aM0
P221‐T	Male	46	Papillary thyroid carcinoma	Primary lesion	Central	T1bN1aM0
P222‐T	Male	47	Papillary thyroid carcinoma	Primary lesion	Central	T1aN1aM0
P223‐T	Female	45	Papillary thyroid carcinoma	Primary lesion	Central	T1aN1aM0
P224‐T	Male	36	Papillary thyroid carcinoma	Primary lesion	Central	T1aN1aM0
P225‐T	Female	56	Papillary thyroid carcinoma	Primary lesion	Central	T1aN1aM0
P226‐T	Female	44	Papillary thyroid carcinoma	Primary lesion	Central	T1aN1aM0
P227‐T	Female	44	Papillary thyroid carcinoma	Primary lesion	Central	T1aN1aM0
P228‐T	Female	70	Papillary thyroid carcinoma	Primary lesion	Central	T1bN1aM0
P229‐T	Female	51	Papillary thyroid carcinoma	Primary lesion	Lateralized	T1aN1bM0
P230‐T	Female	31	Papillary thyroid carcinoma	Primary lesion	Lateralized	T1aN1bM0
P231‐T	Male	41	Papillary thyroid carcinoma	Primary lesion	Lateralized	T1bN1bM0
P232‐T	Male	41	Papillary thyroid carcinoma	Primary lesion	Lateralized	T2N1bM0
P233‐T	Female	35	Papillary thyroid carcinoma	Primary lesion	Lateralized	T1aN1bM0
P234‐T	Female	29	Papillary thyroid carcinoma	Primary lesion	Lateralized	T1bN1bM0
P235‐T	Female	53	Papillary thyroid carcinoma	Primary lesion	Lateralized	T1bN1bM0
P236‐T	Male	32	Papillary thyroid carcinoma	Primary lesion	Lateralized	T1bN1bM0
P237‐T	Female	25	Papillary thyroid carcinoma	Primary lesion	Lateralized	T1bN1bM0
P238‐T	Female	30	Papillary thyroid carcinoma	Primary lesion	Lateralized	T2N1bM0
P239‐T	Female	33	Papillary thyroid carcinoma	Primary lesion	Lateralized	T2N1bM0
P240‐T	Female	36	Papillary thyroid carcinoma	Primary lesion	Lateralized	T1aN1bM0
P241‐T	Male	51	Papillary thyroid carcinoma	Primary lesion	Lateralized	T1aN1bM0
P242‐T	Male	37	Papillary thyroid carcinoma	Primary lesion	Lateralized	T1bN1bM0
P243‐T	Male	30	Papillary thyroid carcinoma	Primary lesion	Lateralized	T1bN1bM0
P244‐T	Female	29	Papillary thyroid carcinoma	Primary lesion	Lateralized	T1aN1bM0
P245‐T	Female	29	Papillary thyroid carcinoma	Primary lesion	Lateralized	T1aN1bM0
P246‐T	Male	14	Papillary thyroid carcinoma	Primary lesion	Lateralized	TxN1bM0
P247‐T	Female	31	Papillary thyroid carcinoma	Primary lesion	Lateralized	T2N1bM0
P248‐T	Female	31	Papillary thyroid carcinoma	Primary lesion	Lateralized	T1aN1bM0
P249‐T	Male	49	Papillary thyroid carcinoma	Primary lesion	Lateralized	T2N1bM0
P250‐T	Male	52	Papillary thyroid carcinoma	Primary lesion	Lateralized	TxN1bM0
P251‐T	Female	31	Papillary thyroid carcinoma	Primary lesion	Lateralized	T1bN1bM0
P252‐T	Female	31	Papillary thyroid carcinoma	Primary lesion	Lateralized	T1bN1bM0

### 3.2 Haematoxylin and eosin staining

Haematoxylin and eosin (H&E) staining was performed using standard protocols. Following deparaffinization and rehydration, sections were stained with haematoxylin solution for 5 min, followed by five consecutive washes with 1% acid ethanol (1% HCl in 70% ethanol). After rinsing with water, sections were stained with eosin for 3 min. Finally, the slides were dehydrated with a graded ethanol series and mounted with a coverslip. The stained slides were examined under a light microscope (Leica DM3000; Leica Microsystems).

#### 3.2.1 Tissue preparation and ST library construction

We collected PTC tumour samples from Ruijin Hospital and subjected them to a rigorous preparation process prior to analysis with 10x Genomics’ Visium Spatial Transcriptomics platform. Initially, tissue samples were dissected into 4–5 mm^3^ pieces, cleaned and snap‐frozen in optimal cutting temperature (OCT) at −80°C. Subsequently, the samples were cryosectioned at a thickness of 10 µm and mounted onto ST arrays. Following dehydration with isopropanol and staining with H&E, the sections were imaged using a 3D HISTECH Pannoramic MIDI FL scanner at 40 × resolution. For library preparation, the Spatial Transcriptomics team employed slides featuring 55 µm diameter spots to capture mRNA from the tissue. During tissue optimization, sections were fixed and permeabilized on Visium Spatial Tissue Optimization Slides to bind mRNA to capture probes. Fluorescently labelled cDNA was synthesized and visualized to determine optimal permeabilization times. The mRNA‐captured cDNA was then spatially barcoded and sequenced, enabling precise mapping of gene expression back to its corresponding tissue location. This approach ensures a direct correlation between each gene expression spot and its source location within the tissue, crucial for understanding the spatial dynamics of gene activity within the tumour microenvironment.

### 3.3 Spatial transcriptome and data analysis

Spatial transcriptomic slides were printed with capture areas from four patients with lymph node metastatic thyroid cancer, the same patients from whom scRNA‐seq data was obtained. Gene expression information for these spatial transcriptomic slides was captured using the Visium Spatial Gene Expression platform (10x Genomics), employing spatially barcoded mRNA‐binding oligonucleotides, as per the manufacturer's instructions. Raw sequencing reads from the spatial transcriptomes were subjected to quality control and mapping. The demultiplexed clean reads were aligned against the UCSC human GRCh38 reference genome using Space Ranger (v. 1.3; 10x Genomics). After obtaining the single‐cell gene expression count matrix, we utilized Seurat v. 4.0 for downstream analysis on the R platform (v. 4.1.2; The R Foundation for Statistical Computing). In addition, we employed SpaGCN, a recently developed graph convolutional network, to differentiate spatial patterns and domains based on DEGs.^14^ In the SpaGCN analysis, the default mode was used to autonomously define domains within the spatial data. Super‐resolution tumour‐edge maps were generated using TESLA.^15^ Finally, integrated single‐cell and spatial data analyses were performed using CellTrek, a computational framework that facilitates the direct mapping of single cells to their spatial coordinates in tissue sections by integrating scRNA‐seq and spatial transcriptomic data. This integration enabled the generation of a cell−cell communication map.^20^

#### 3.3.1 Single‐cell sequencing experiments

Collected PTC tumour tissues were processed into single‐cell suspensions for single‐cell RNA sequencing. Initially, tissues were washed thrice with cold Dulbecco's Phosphate‐Buffered Saline (DPBS, Gibco) following dissection, then incubated in a pre‐warmed digestion buffer containing 2 mg/mL collagenase I and II, .9 U dispase, trypsin and Dulbecco's Modified Eagle Medium (DMEM) at 37°C. The tissues were subsequently minced into smaller pieces and gently agitated on a 37°C heat block for 15 min. A small aliquot (10 µL) of the resulting suspension was examined under a microscope using a haemocytometer to assess cell dissociation. The cell suspension was then filtered through a 70‐µm cell strainer and washed with 5 mL DMEM. Cells were concentrated by centrifugation at 500 × *g* for 5 min at 4°C. The supernatant was discarded, and the cell pellet was resuspended in 50–100 µL DMEM supplemented with 10% DPBS. Cell density was adjusted to 700–1200 cells/µL prior to loading onto the 10x Genomics Chromium system. Gel Bead‐In‐EMulsions were formed, facilitating reverse transcription and barcode introduction. Following reverse transcription, first‐strand cDNA was isolated using magnetic beads. After quality control and quantification, the cDNA was used to construct a library with the 10 × Chromium Single Cell 5' Reagent Kits (v2, 10x Genomics) and sequenced on the Illumina NovaSeq platform.

### 3.4 ScRNA‐seq analysis

Single‐cell sequencing data were aligned and barcode‐demultiplexed using the Cell Ranger v. 3.0.2 vdj pipeline (10x Genomics). The data were filtered based on quality control criteria: cells with fewer than 800 detected genes, genes detected in fewer than 5 cells per dataset and cells with mitochondrial gene expression exceeding 10% of the total expression level were excluded. Potential doublets were removed by excluding cells with the top 5% of total transcript unique molecular identifiers (UMIs). Scrublet was also employed to further identify and remove doublets. All datasets were combined, and the top 2000 highly variable genes were selected. To account for technical variations, the total UMI counts and mitochondrial gene expression proportions were regressed out for each cell. The data was scaled, and PCA was performed to reduce dimensionality. Batch correction was applied using the BBKNN method with ‘donor’ as the batch key. These preprocessing steps were conducted using the Scanpy framework.

Seurat was employed for downstream analysis. Initial clustering was performed using the FindClusters function with a resolution of .1 to identify major cell types. For subsequent sub‐clustering of immune cells, the resolution was set to .3. Known marker genes were utilized to annotate the clusters. CopyKat was employed to infer cell ploidy on a randomly down‐sampled dataset.^21^ To manage computational demands, cells were down‐sampled to 2000 cells per cluster before running this analysis. Default parameters were used for CopyKat (ngene.chr = 5, win.size = 25, KS.cut = .1). To validate CopyKatt results, we also applied the InferCNVsoftware. Using InferCNV version 1.12.0, we down‐sampled the data to select 7000 cells, ensuring equal representation of 1000 cells from each primary cell type. This process resulted in the retention of 26 428 features for subsequent analysis with InferCNV. Differential gene expression was determined using the FindMarkers or FindAllMarkers function with a fold‐change threshold of .25 and gene detection rate threshold (min.pct) set to .25. Only genes with an adjusted *p*‐value less than .05 were considered differentially expressed. Gene set enrichment analysis was conducted using databases incorporated in the enrich R package. Pseudo‐time analysis was performed using Monocle with default settings.^22^ The log fold change threshold was set to .5. Cell−cell interaction analysis was performed with the R package CellChat.^23^

### 3.5 Immunofluorescence

Standard immunostaining techniques were employed. Briefly, sections were permeabilized with .5% Triton X‐100 in phosphate‐buffered saline (PBS) for 10 min at room temperature and blocked with serum‐free protein‐blocking solution (Dako; Agilent Technologies) for 60 min. For colocalization staining, slides were sequentially incubated with primary antibodies. After incubation with the secondary antibody, sections were stained with 4',6‐diamidino‐2‐phenylindole (DAPI) for nuclear visualization. The sections were imaged using a confocal laser‐scanning fluorescence microscope (ZEISS). ImageJ software was used for quantitative analysis. The following antibodies were utilized: anti‐CK19 (MAB‐0829, MXB), anti‐*APOE* (Abcam Cat#ab51015) and anti‐Gr‐1 (Abcam Cat#ab238132).

### 3.6 Cell culture and transfection

Hth‐7 and TPC‐1 cell lines were purchased from Procell Company (BCRJ Cat# 0397, RRID:CVCL_6298). Cells were cultured in DMEM supplemented with 10% foetal bovine serum (FBS) and 1% penicillin‐streptomycin at 37°C in a 5% CO_2_ atmosphere. *APOE* overexpression control and lentiviral vectors were purchased from GenePharma. TPC‐1 and Hth‐7 cells were seeded in six‐well plates 24 h prior to transfection with viruses and transfection reagent (RNAi‐Mate, GenePharma). *APOE*‐overexpressing cell lines were selected and cultured in a medium containing 5 µg/mL puromycin.

### 3.7 RNA isolation and qPCR

Total cellular RNA was extracted using an RNA extraction kit (Takara Bio). The extracted RNA was reverse transcribed into cDNA using the Vazyme reverse transcription kit. The resulting cDNA was subjected to quantitative‐PCR (qPCR) using the SYBR Green kit (Vazyme). qPCR reactions were performed on a CFX96 Touch Real‐Time PCR Detection System (Bio‐Rad Laboratories, RRID:SCR_008426). The primers used for qPCR were as follows: βactin‐forward: 5′‐CATGTACGTTGCTATCCAGGC‐3′; βactin‐reverse: 5′‐CTCCTTAATGTCACGCACGAT‐3′. *APOE*‐forward: 5′‐ GTTGCTGGTCACATTCCTGG‐3′; *APOE*‐reverse: 5′‐GCAGGTAATCCCAAAAGCGAC‐3′. The relative expression levels were calculated using the 2^‒ΔΔCt^ method.

### 3.8 Cellular proliferative ability

The TPC‐1 and Hth‐7 cells were seeded in 96‐well plates at a density of 2000 cells per well and incubated for 24 h. A CCK‐8 assay (Vazyme) was performed according to the manufacturer's instructions at 37°C in 5% CO_2_. The CCK‐8 reagent was added to each well and incubated for 2 h at 37°C. Optical density was measured at 450 nm using a SpectraMax 190 microplate reader (Molecular Devices).

### 3.9 Colony formation assays

We seeded 200 TPC‐1 and Hth‐7 cells per well into 6‐well plates and replaced the medium every 3–4 days. After a 2‐week incubation period, cells were fixed with 4% paraformaldehyde, washed three times with PBS and stained with .05% crystal violet for 30 min. The plates were then washed, and colonies containing more than 50 cells were photographed and counted.

### 3.10 Transwell migration and invasion assays

Transwell chambers (8‐µm pores; Corning) pre‐coated with Matrigel were employed for the invasion assay. Cells (2 × 10^5^ cells/well) were cultured in the upper chamber in 200 µL of DMEM supplemented with 5% FBS. In the lower compartment, 700 µL of DMEM containing 20% FBS was added. After a 24‐h incubation period, cells were fixed with 4% paraformaldehyde, washed thrice with PBS and stained with .05% crystal violet for 30 min. Cells on the inner surface of the chamber were removed using sterilized cotton swabs. The cells were subsequently examined under a light microscope (Leica DM3000; Leica Microsystems). ImageJ software (ImageJ, RRID:SCR_003070) was utilized for quantification.

#### 3.10.1 Animals

All mice were housed in specific pathogen‐free conditions and fed a standard chow diet. C57 mice were obtained from the Model Animal Research Center at Nanjing University, China. We generated APOE‐knockout mice using traditional CRISPR/Cas9 techniques. Briefly, we identified target sites for sgRNA using an online CRISPR design tool (http://zlab.bio/guide‐design‐resources) and designed sgRNA oligos targeting the *APOE* gene according to CRISPR/Cas9 system guidelines. In vitro synthesized RNA was then combined with Cas9 protein to form gRNA‐Cas9 complexes. Purified gRNA and Cas9 mRNA were injected into mouse zygotes. All animal procedures were approved by the Ethics Committee for Animal Experiments at Shanghai Jiao Tong University School of Medicine, ensuring adherence to ethical guidelines.

### 3.11 Xenotransplantation model

Female BALB/c nude mice, aged 3–4 weeks and weighing 13–15 g, were obtained from Shanghai Phenotek Laboratory Animal Co., Ltd. Tumour models were established by subcutaneously injecting1 × 10⁷TPC‐1 and Hth‐7 cells, suspended in Matrigel/PBS, into the left hind limbs of nude mice. Tumour size and mouse weight were monitored every 3–4 days. Tumour volume was calculated using the formula: .5 × length × width × width. After 2 weeks, mice were euthanized, and tumours were harvested, photographed and fixed with 4% paraformaldehyde. All animal studies were approved by the Animal Use Committee of Shanghai Jiao Tong University (SYXK 2018‐0027).

### 3.12 Immunohistochemistry

After dewaxing, samples were microwaved in ethylene diamine tetra acetic acid buffer and blocked for 60 min with a serum‐free protein‐blocking Solution (Agilent Technologies). Primary antibodies against *APOE* and *ABCA1* (Abcam; 1:500 dilution) were applied to the sections and incubated overnight at 4°C. Subsequently, the tissues were incubated with secondary antibodies for 1 h at room temperature. The sections were then imaged using a confocal laser‐scanning fluorescence microscope (ZEISS). ImageJ software (RRID:SCR_003070) was employed for quantification.

#### 3.12.1 mRNA‐seq experiments and analysis

Two cell lines, one with *APOE* overexpression and the other without, were harvested in triplicate for RNA extraction. Paired‐end sequencing libraries were prepared using the ABclonal mRNA‐seq Lib Prep Kit according to the manufacturer's protocol. Starting with 1 µg of total RNA, mRNA was isolated using oligo (dT) beads, fragmented and reverse transcribed into first‐strand cDNA using hexamer primers and Reverse Transcriptase (RNase H). Second‐strand cDNA synthesis followed. The resulting double‐stranded cDNA was subjected to adapter ligation and PCR amplification. The final library was purified and quality‐controlled using the Agilent Bioanalyzer 4150. Sequencing was performed on an Illumina NovaSeq6000 (or MGISEQ‐T7), generating 150 bp paired‐end reads.

The initial processing pipeline involved cleaning raw fastq reads using custom Perlscripts. These scripts eliminated adapter sequences and discarded low‐quality reads with either a high proportion (over 60%) of bases having a low‐quality score (under 25) or a significant amount (more than 5%) of ‘N’ bases, which represent uncertain base information. The resulting clean reads were then aligned to a reference genome using Kallisto.^24^ To identify DEGs, DESeq2 software (http://bioconductor.org/packages/release/bioc/html/DESeq2.html) was employed.^25^ Genes with an absolute log2 fold change exceeding 1 and an adjusted *p*‐value less than .05 were deemed significantly differentially expressed. Finally, a well‐established gold standard pipeline was utilized for gene set enrichment analysis.^26^

#### 3.12.2 Sample preparation and LC‐MS/MS‐based metabolomics analysis

Cell samples for metabolomics analysis were prepared as previously reported for LC‐MS.^27^

Briefly, samples were fully ground under dry ice conditions, and the resulting mixture was concentrated to dryness via centrifugation. The sample was then reconstituted and centrifuged, and the supernatant was collected for LC‐MS/MS analysis. LC‐MS/MS was employed to monitor metabolite detection and quantification. The raw data underwent peak alignment, retention time correction and peak area extraction using the Compound Discovery program. Metabolite structures were identified based on accurate mass number matching (< 10 ppm) and second‐order spectrogram matching. R statistical software version 2.15.0 was utilized to perform multidimensional statistical analysis, including PCA, partial least squares‐discriminant analysis and orthogonal partial least squares‐discriminant analysis.

#### 3.12.3 Flow cytometry

The spleens of C57BL/6 and *APOE* ^−^/^−^ mice were aseptically harvested, mechanically and enzymatically dissociated into single‐cell suspensions, and subjected to red blood cell lysis. Immune cell counts were determined using a cell counter. Following antibody staining, flow cytometry was employed to quantify mature DCs (CD45^+^CD11^+^CD80^+^CD86^+^), NK (CD11^+^CD3^−^NK1.1^+^), NKT (CD11^+^CD3^+^NK1.1^+^), M1 macrophage (CD45^+^CD11^+^CD86^+^), M2 macrophage (CD45^+^CD11^+^CD206^+^), CD4^+^T (CD45^+^CD3^+^CD4^+^) and CD8^+^T (CD45^+^CD3^+^CD8^+^). All antibodies were sourced from BD Biosciences.

#### 3.12.4 Machine learning and statistical analysis

To develop a metastasis prediction model, we utilized thyroid tumour cells labelled as positive or negative based on patient phenotype. Gene expression served as features for prediction, with the feature space to the top 2000 highly variable genes and then ranked by mutual information with the labels using sklearn's SelectKBest function. A random forest classifier (100 trees, minimum three samples per leaf) was constructed, optimized by scanning feature numbers from 2 to 20 and assessed through five‐fold cross‐validation. Model performance was evaluated primarily using AUROC, along with recall and precision scores. The optimal model was selected based on the smallest feature set that achieved a performance plateau. A similar methodology was employed for training models for central or cervical metastasis, with distinct labelling based on patient phenotypes, excluding non‐metastatic samples. For in vivo and in vitro group comparisons, a Student's *t*‐test was performed. For TCGA survival analysis, Kaplan−Meier curves were computed and visualized to illustrate patient survival rates. Differences in survival across various categories were analysed using the log‐rank test. Additionally, univariate Cox proportional hazards regression was employed to evaluate the hazard ratios associated with each categorical variable.

#### 3.12.5 Quantitative PCR analysis of clinical samples for machine learning (ML)‐based prediction validation

A total of 203 thyroid cancer patients who had undergone thyroid surgery and had complete pathological data were selected for this study, and their clinical information descriptions are provided in Table 3. RNA was extracted from obtained by puncturing the thyroid cancer tissues, which were pathologically confirmed PTC tissues by a needle biopsy technique. The extracted RNA was subsequently reverse transcribed, followed by quantitative real‐time polymerase chain reaction (qRT‐PCR) to evaluate the accuracy of the machine learning models.

## 4 DISCUSSION

To our knowledge, this is the first scRNA‐seq analysis of metastatic PTC that integrates spatial information. By combining scRNA‐seq and spatial transcriptomics, we identified a group of thyroid cancer cells with relatively low *APOE* expression. According to the TCGA dataset, *APOE* expression was negatively correlated with overall survival. Our cell line experiments and nude mouse tumorigenicity assays further indicated that *APOE* overexpression inhibited tumour cell proliferation and migration. While *APOE* is primarily known for its role in lipid metabolism,^28^ recent studies have highlighted its involvement in cell proliferation, tumour angiogenesis and metastasis.^29^, ^30^, ^31^, ^32^ Consistent with our findings, two previous studies using TCGA data reported an association between low *APOE* expression and older age, advanced TM stage and shorter overall survival in PTC patients.^29^, ^32^ *APOE* expression also varies among patients based on race, age, cancer stage, nodal metastases and histological subtype.^29^, ^30^ In our study, we identified *APOE* ^−^ tumour cells in 14 patients with advanced PTC at the single‐cell level and elucidated their role in a large tumour‐immune cell communication network. Overexpression of *APOE* in tumour cell lines reversed their proliferative phenotype in both cell and animal experiments. Additionally, we found that *APOE* overexpression influenced multiple pathways related to metabolic remodelling and immunity, which are crucial for tumorigenesis and cancer progression. Therefore, we propose that *APOE* expression can serve as a valuable biomarker for metastatic thyroid cancer.

Although thyroid cancer is generally indolent, metastases to adjacent lymph nodes are common and can lead to a more advanced disease state. Lymphatic metastases primarily occur in the central region, followed by the lateral region, with cervical lymph node involvement being associated with a poorer prognosis.^33^, ^34^, ^35^ Compared to their counterparts in central compartment lymph nodes, thyroid cancer cells in cervical LNMs exhibited significantly lower *APOE* expression. A similar role of *APOE* expression has been identified in the metastases of melanoma cells and non‐small cell lung cancer.^17^, ^36^, ^37^ *APOE* reportedly functions as a metastasis‐suppressive protein by inhibiting both the invasiveness of melanoma cells and the recruitment of endothelial cells, thus serving as a barrier to metastatic colonization.^17^, ^37^ An et al.^36^ also discovered a negative association between LNM and *APOE* staining intensity in small, preoperative biopsies of non‐small cell lung cancer patients. They further suggested that *APOE* might contribute to a metastasis‐inhibiting tumour microenvironment by modulating inflammatory factors and anticancer T cells.

Our data further indicated that thyroid tumour cells with low *APOE* expression exhibited significantly decreased overall intercellular communication with immune cells, particularly NK and T cells. A separate bioinformatics analysis of *APOE* in PTC tissues revealed that genes co‐expressed with *APOE* were functionally enriched in adaptive immune response, cell chemotaxis and transcriptional misregulation.^29^ Moreover, *APOE* expression levels were correlated with tumour‐infiltrating immune cells and immune biomarkers in thyroid cancer.^29^ Regarding *APOE*’s role in immune modulation, Tavazoie et al.^17^ demonstrated that *APOE*, as a transcriptional target of liver X receptors (*LXR*s), can impair the survival and abundance of immunosuppressive MDSCs, both in vitro and in vivo. Conversely, *APOE* inactivation can impair immunity through MDSC accumulation. Our RNA‐seq data from *APOE*‐overexpressing thyroid cancer cell lines showed increased expression of *ABCA1*, a cofactor in *LXR* activation, suggesting that *APOE* overexpression may induce *LXR*, thereby potentially rescuing impaired immunity in tumours. By suppressing *LXR, APOE* may influence immunity by promoting MDSC accumulation in PTC. Consequently, *LXR*−*APOE* interactions could modulate the tumour microenvironment, potentially impacting immune cell composition.

With the advantage of an integrated spatial transcriptome, we also discovered that thyroid tumour cells with low *APOE* expression interacted with DCs and CD4^+^ T cells via CD99‐regulated signalling but minimally interacted with the *CD6* receptor. *CD99* and *CD6* are type I integral transmembrane glycoproteins broadly expressed in various cell types. *CD99* isoforms have been reported to have opposing functions in mediating inflammation, T cell regulation, tumour cell invasion and migration.^38^, ^39^, ^40^ *CD6*, in conjunction with its two known ligands (*CD166* and *CD318*), is primarily responsible for adhesive contacts between T cells and antigen‐presenting cells, and subsequent proliferative and differentiative responses.^41^, ^42^ The observed discrepancies in *CD99* and *CD6* binding in *APOE*‐low thyroid tumour cells contribute to oncogenic functions, although the underlying mechanisms require further investigation. To explore the influence of *APOE* on immunity, we employed CRISPR/Cas9 gene editing technology. We found that while CD4^+^ and CD8^+^ T cell numbers remained unchanged, DC, NK and NKT cell abundance decreased in *APOE*‐knockout mice. Additionally, the proportion of peritoneal M1/M2 macrophages was attenuated in these mice.

Meanwhile, some studies have drawn conclusions contrary to ours.^31^, ^43^, ^44^, ^45^ Huang et al.^31^ utilized the fat‐mass and obesity‐associated protein (FTO) to epigenetically inhibit *APOE* expression in PTC, leading to the inhibition of glycolytic metabolism via the *IL‐T/JAK/STAT3* signalling pathway and subsequent suppression of tumour growth. Additionally, *APOE* has been reported to be overexpressed in several other malignancies, including bladder, breast and gastric cancer,^43^, ^44^, ^45^ and has been significantly correlated with tumour staging and LNM in pancreatic and endometrial cancer.^46^, ^47^ These discrepancies may be attributed to the existence of different *APOE* isoforms, as distinct isoforms could exert either protective or inhibitory effects on cancer development.^48^, ^49^ Further research is necessarily warranted to elucidate the specific impact of different *APOE* isoforms on thyroid cancer progression and metastasis. This conclusion is supported by evidence from other studies identified in our search. However, our study has not fully explored the precise molecular mechanisms underlying *APOE*’s influence on the tumour immune microenvironment. Future investigations are required to delve deeper into this impact. Simultaneously, probing the unique role of *APOE* in PTC is crucial for future clinical translation.

We also developed a machine‐learning bioinformatics model based on scRNA‐seq data from in‐situ thyroid cancer tissues to establish a 13‐gene signature predictive of LNM. Our results were validated through qPCR, achieving a specificity and sensitivity of 90%. To our knowledge, only one previous machine learning‐based study has investigated molecular biomarkers of thyroid cancer progression, identifying a 25‐gene panel predictive of LNM with a sensitivity of 86% and a specificity of 62%.^50^ Compared to this previous study, our 13‐gene panel demonstrated improved predictive accuracy. Our panel was capable of predicting metastatic lymphadenopathy and even recurrence in patients with early‐stage, in‐situ thyroid cancer. This panel may aid oncologists in avoiding unnecessary fine‐needle aspiration and in determining appropriate subsequent treatment.

## 5 CONCLUSION

This study highlights the significance of *APOE* expression as a key characteristic of metastatic thyroid cancer. We observed a reduction in overall intercellular communication with immune cells, including DCs, NK cells and NKT cells, as well as a decrease in the proportion of peritoneal M1/M2 macrophages in *APOE*‐KO mice. *LXR*−*APOE* interactions can modulate the tumour microenvironment, potentially influencing immune cell composition. In summary, *APOE* promotes the proliferation, tumorigenic potential, migration and invasion capabilities of PTC cells by regulating the tumour immune microenvironment.

## AUTHOR CONTRIBUTIONS

Guohui Xiao wrote the main manuscript text. Rongli Xie, Jianhua Gu and Min Ding provided the study materials, reagents, patients, laboratory samples, animals, instrumentation, computing resources and other analysis tools. Yishu Huang performed experiments and analysis of the data. Dongjie Shen, Jiqi Yan and Jianming Yuan formulated the overarching research goals and aims. Qiong Yang and Wen He collected the specimens and patient clinical information. Siyu Xiao collated the data for analysis. Jian Wu provided pathological instructions. Jian Fei, Dan Xu and Haizhen Chen played the oversight and leadership responsibility for the research activity planning and execution, including mentorship external to the core team. All authors reviewed the manuscript.

## CONFLICT OF INTEREST STATEMENT

The authors declare no conflict of interest.

## ETHICS APPROVAL AND CONSENT TO PARTICIPATE

All human tissue samples obtained for this study were approved by the Human Ethics Committee of Ruijin Hospital, Shanghai Jiao Tong University School of Medicine, Shanghai, China (2023LLSDN11).

## CONSENT FOR PUBLICATION

An exemption of signing an informed consent was given by the Ruijin Hospital committee given the retrospective study design.

## Supporting information



Supporting information

Supporting information

Supporting information

Supporting information

Supporting information

Supporting information

Supporting information

Supporting information

Supporting information

Supporting information

Supporting information

Supporting information

Supporting information

Supporting information

## Data Availability

The datasets generated and/or analysed during the current study are available in the Gene Expression Profiling Interactive Analysis 2 (GEPIA2) online tool (http://gepia2.cancer‐pku.cn) (Gene Expression Profiling Interactive Analysis, RRID:SCR_018294).  R code is available upon request. All scRNA‐seq data and ST data were uploaded to https://ngdc.cncb.ac.cn/gsa‐human/ with the project number: PRJCA021247.
