# Supplementary figures and images for: Single‐cell RNA‐sequencing and spatial transcriptomic analysis reveal a distinct population of APOE − cells yielding pathological lymph node metastasis in papillary thyroid cancer
Source: Clin Transl Med. 2025 Jan 15;15(1):e70172. doi: 10.1002/ctm2.70172 (PMC11733439; doi:10.1002/ctm2.70172)

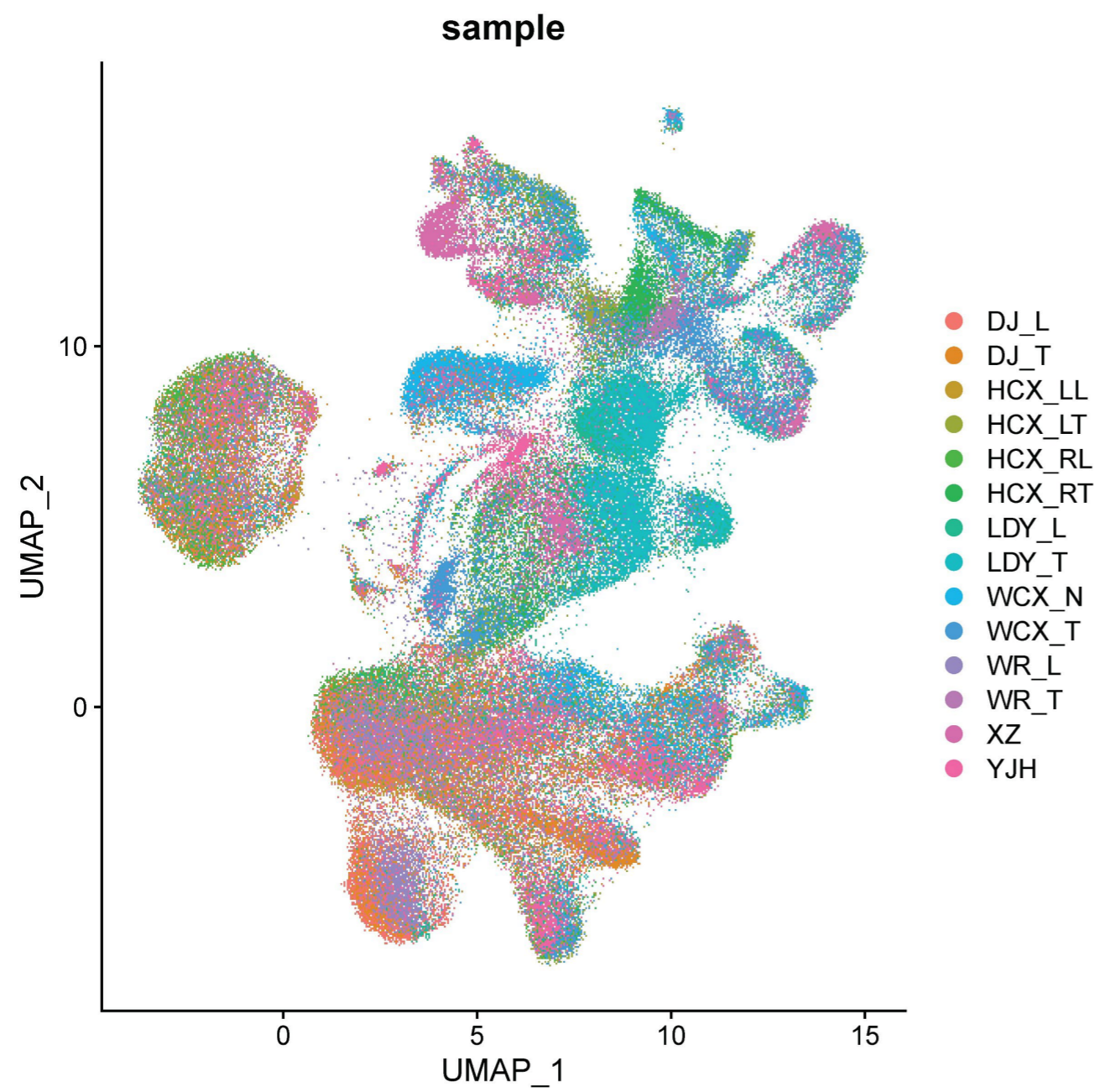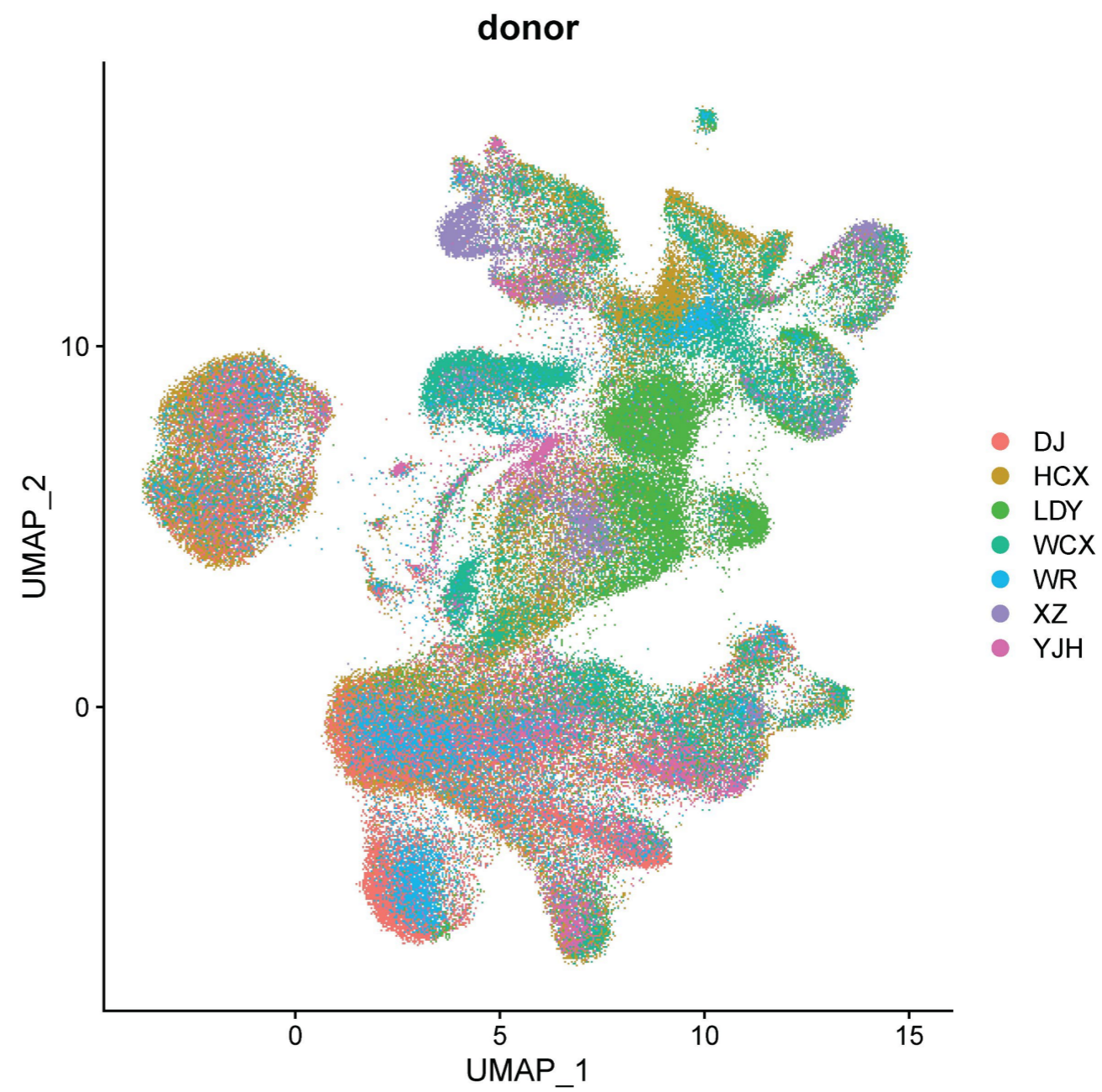

Supplement: Supplementary file 1 — Supporting information [file CTM2-15-e70172-s012.pdf]

inferCNV

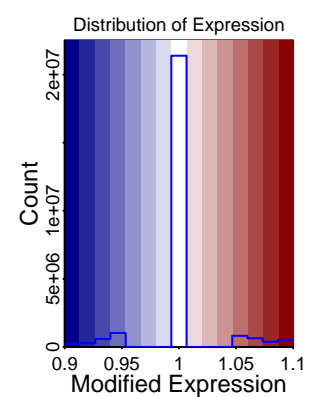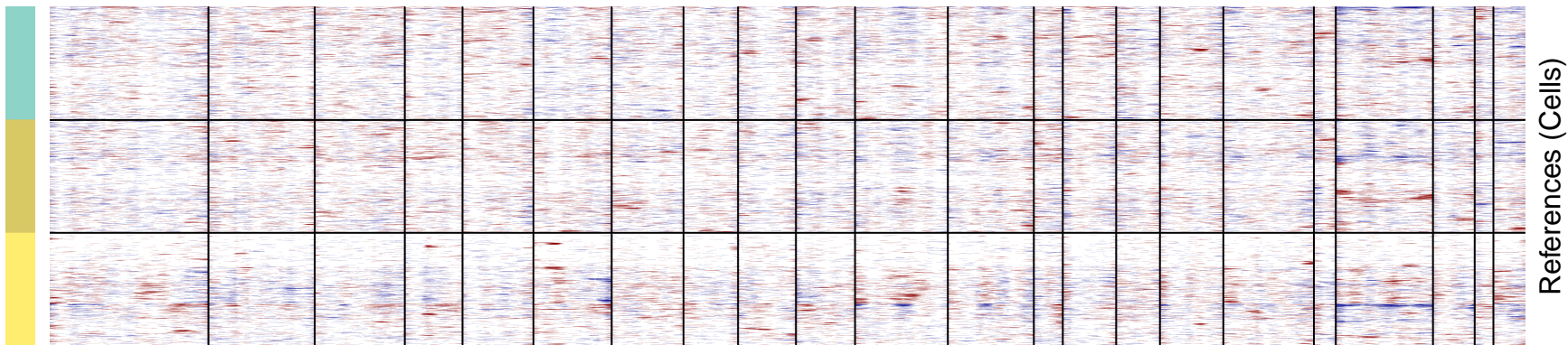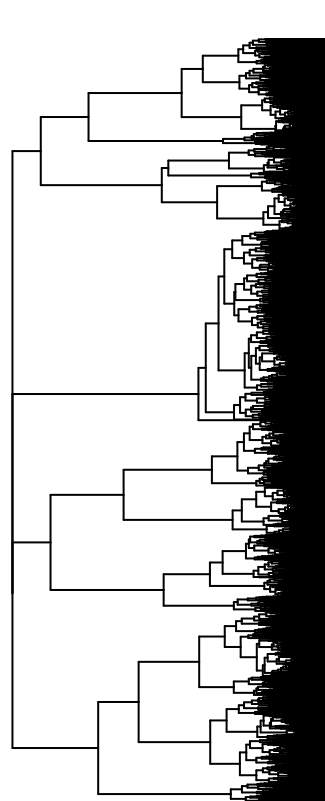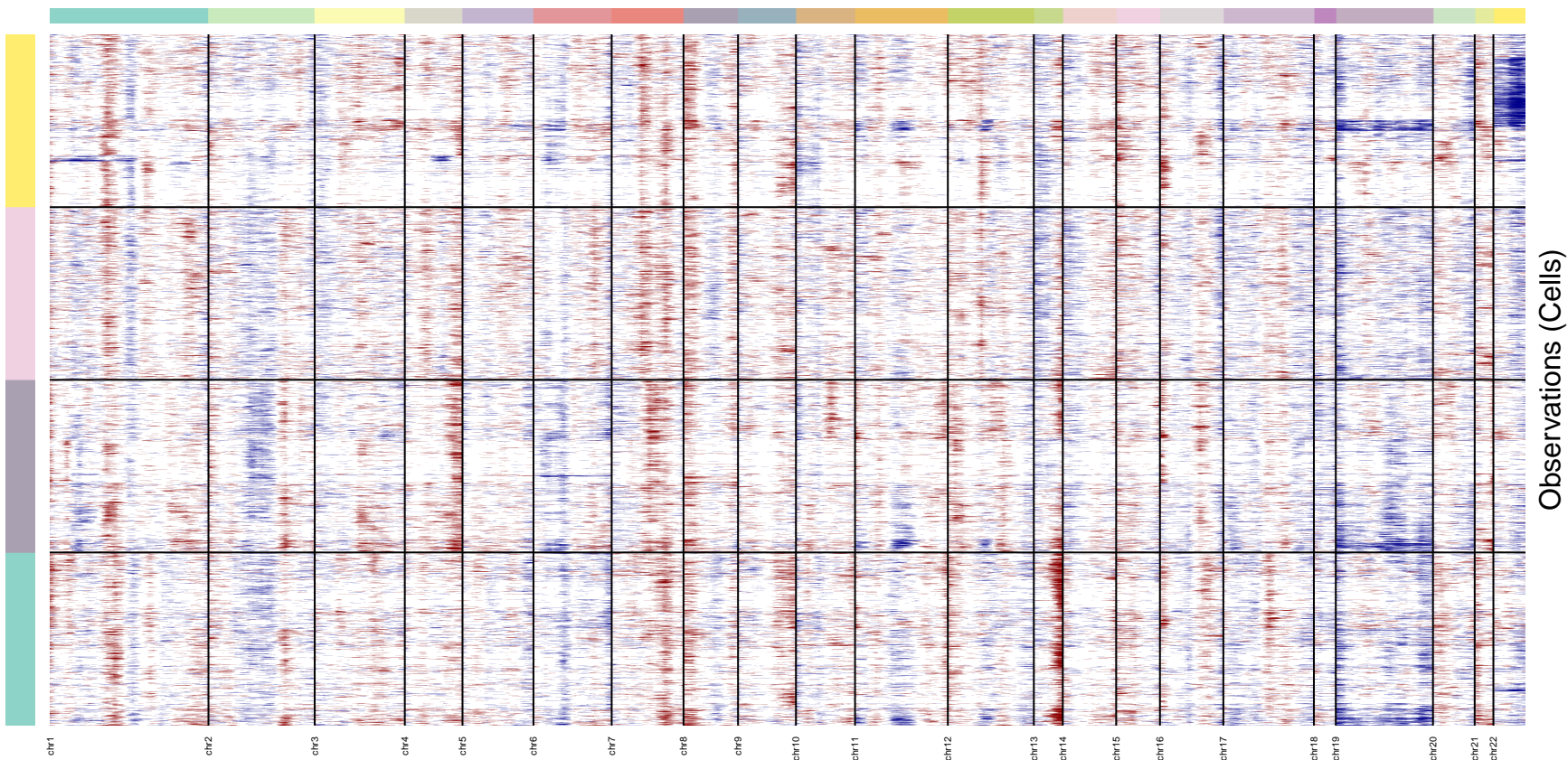

Genomic Region

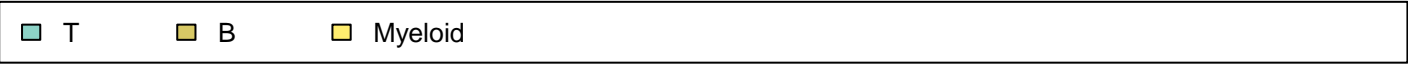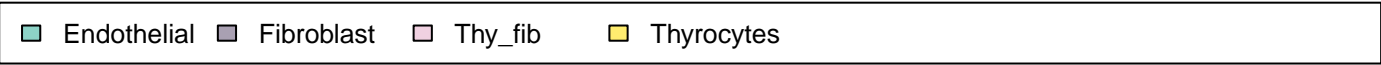

Supplement: Supplementary file 2 — Supporting information [file CTM2-15-e70172-s011.pdf]

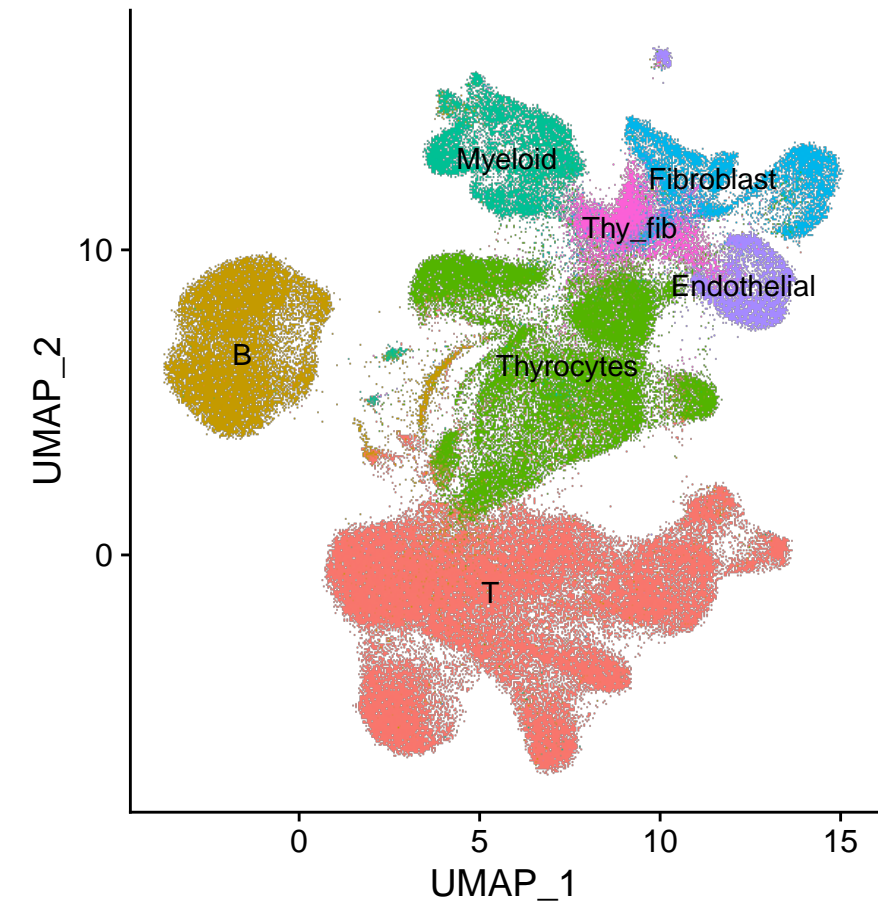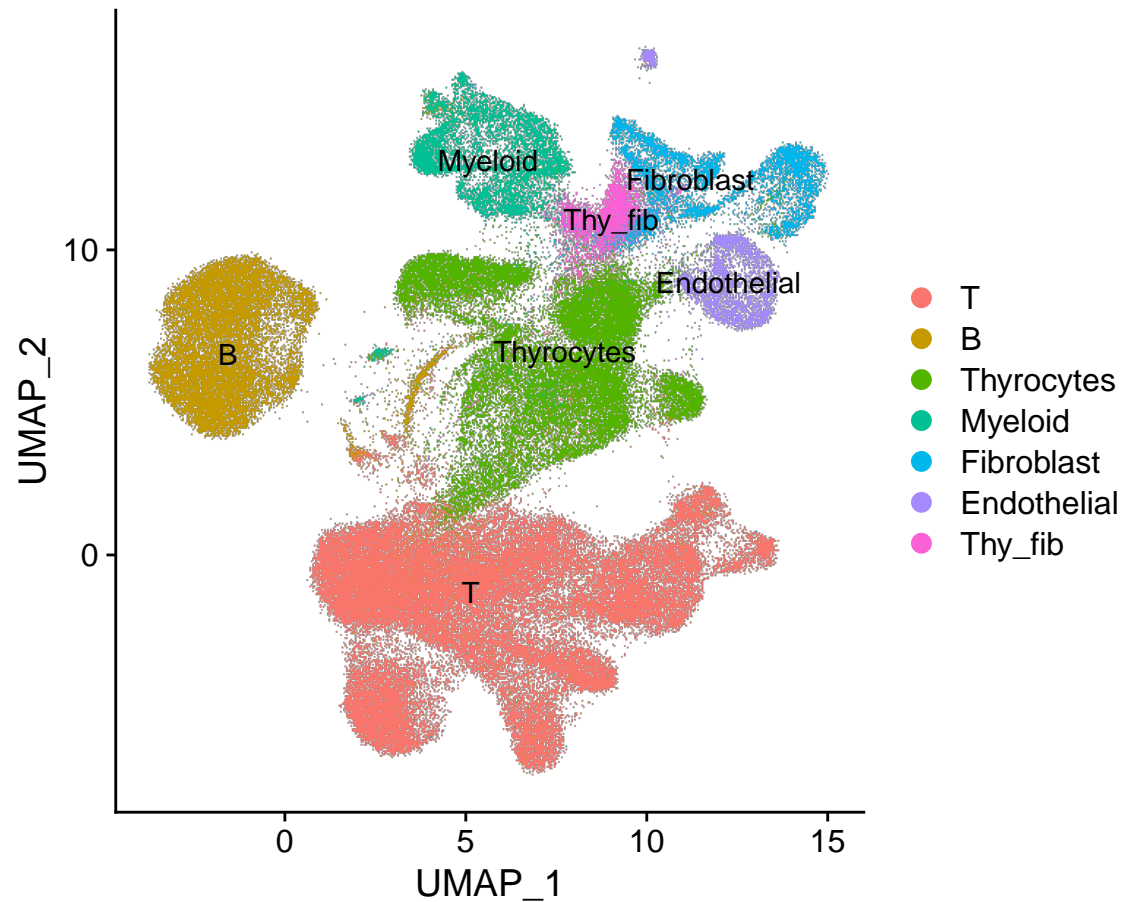

Supplement: Supplementary file 3 — Supporting information [file CTM2-15-e70172-s009.pdf]

A

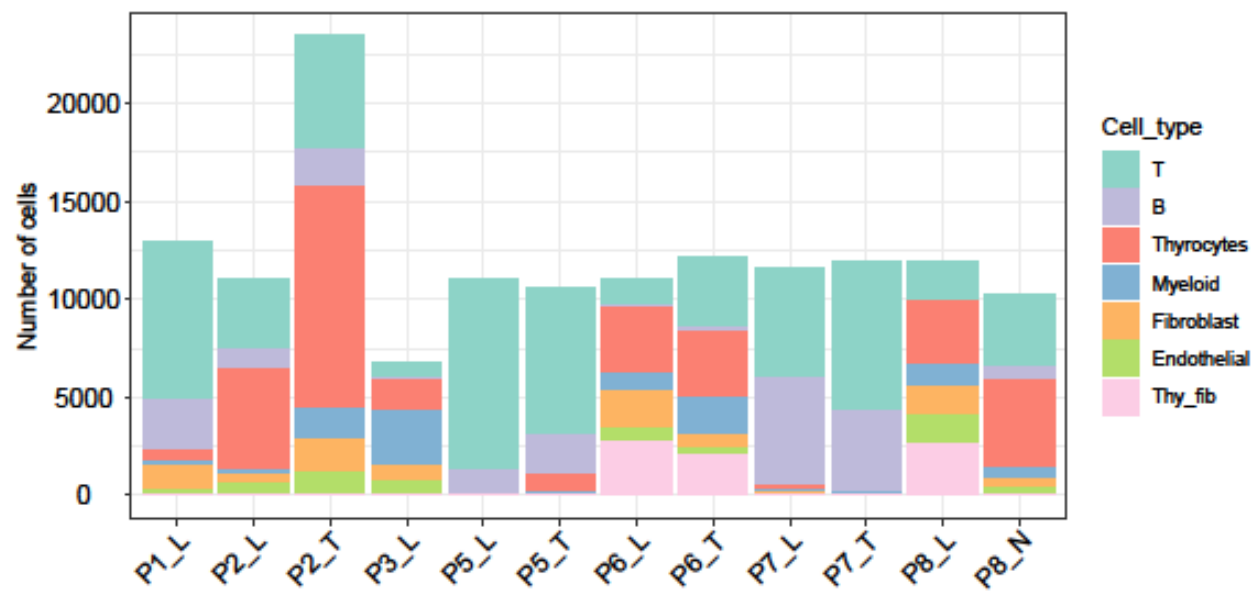

B

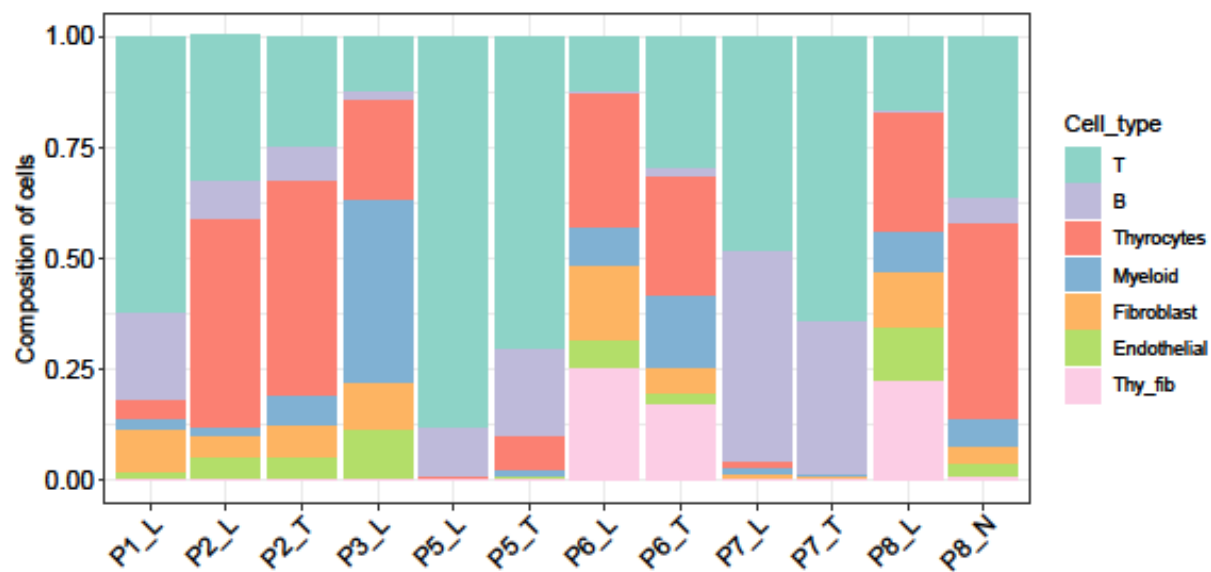

Supplement: Supplementary file 4 — Supporting information [file CTM2-15-e70172-s004.pdf]

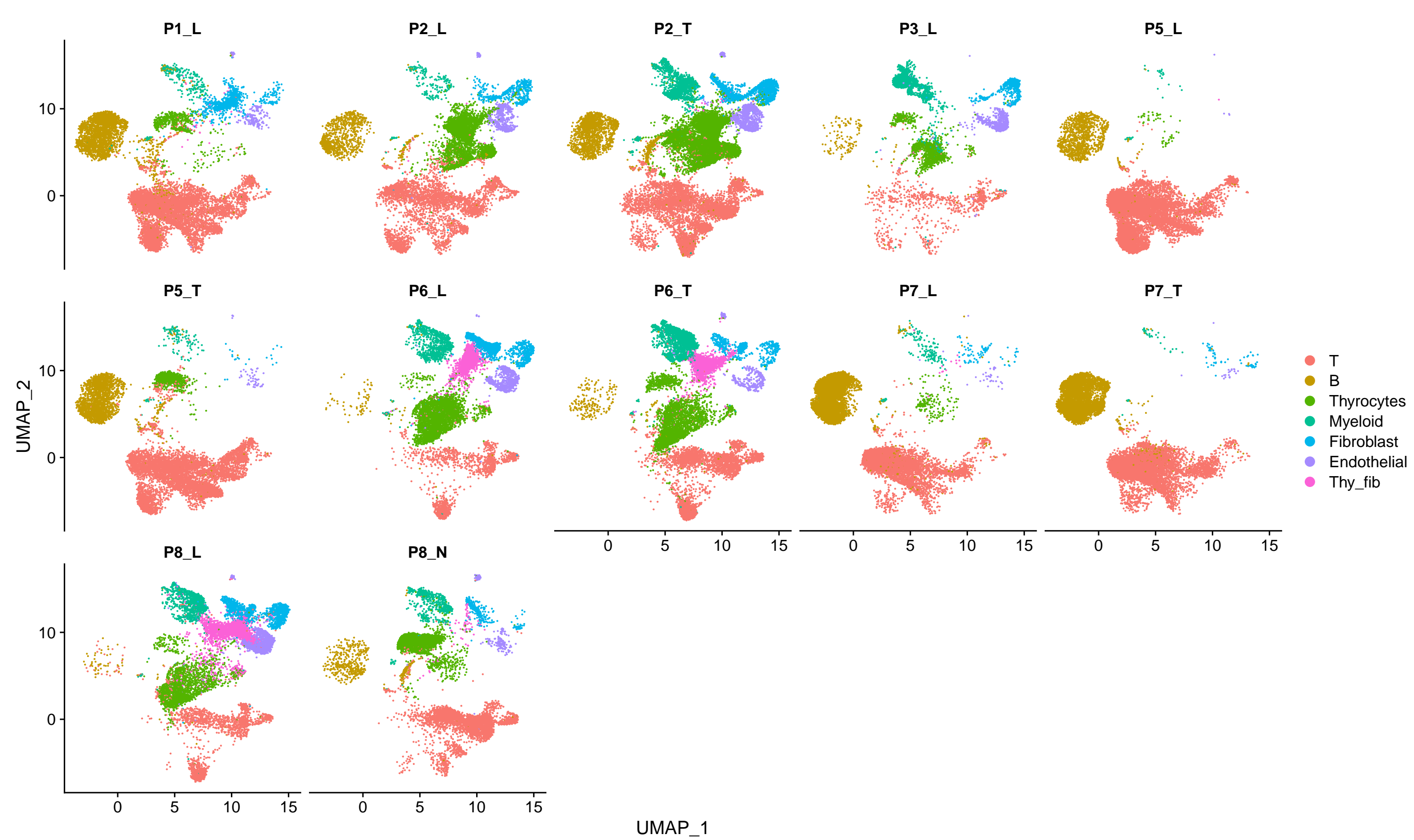

Supplement: Supplementary file 5 — Supporting information [file CTM2-15-e70172-s013.pdf]

P5-L

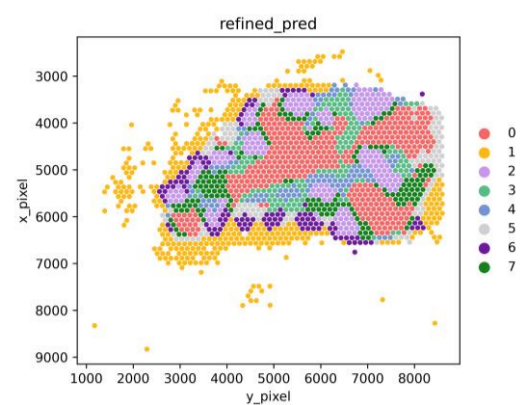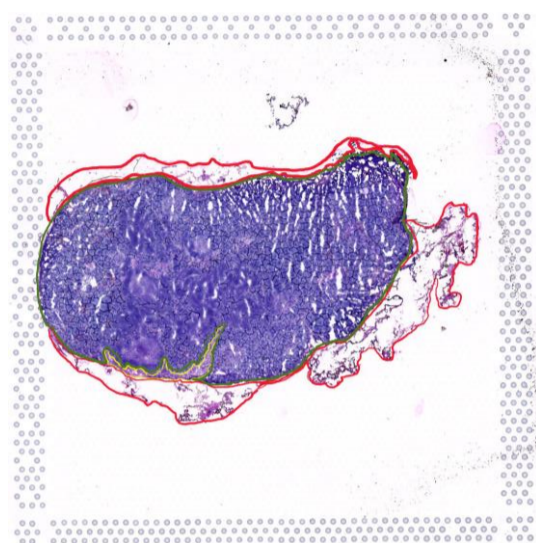

P5-T

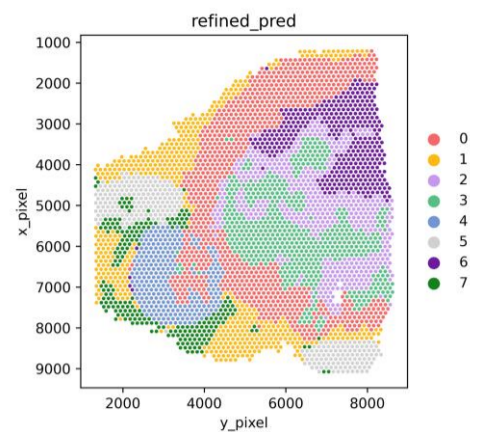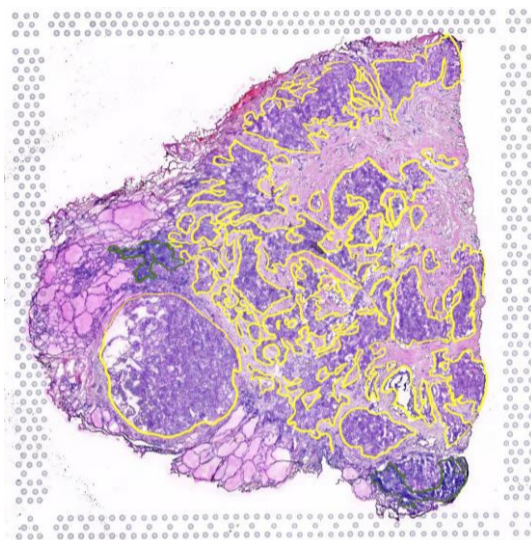

P6-L

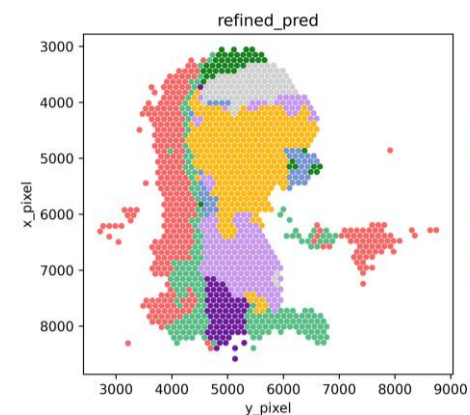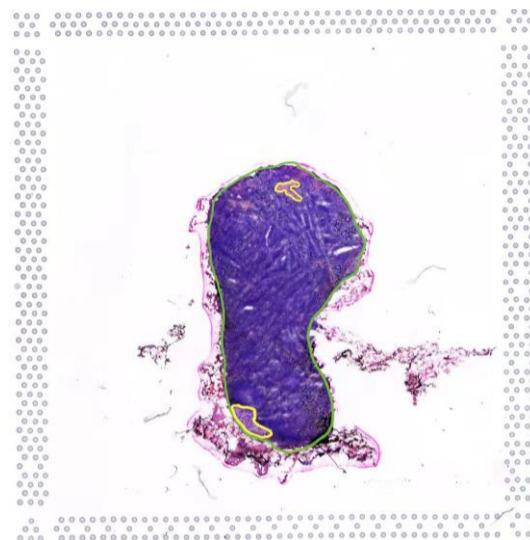

P6-T

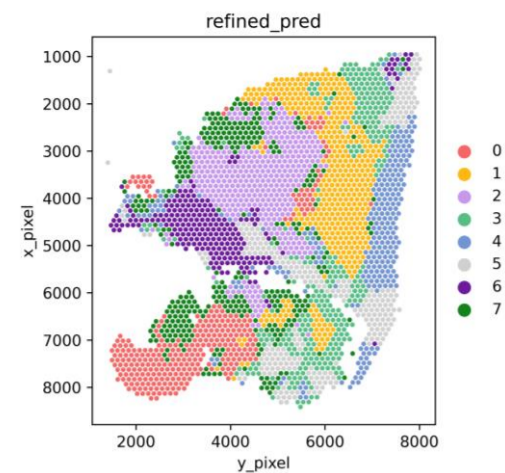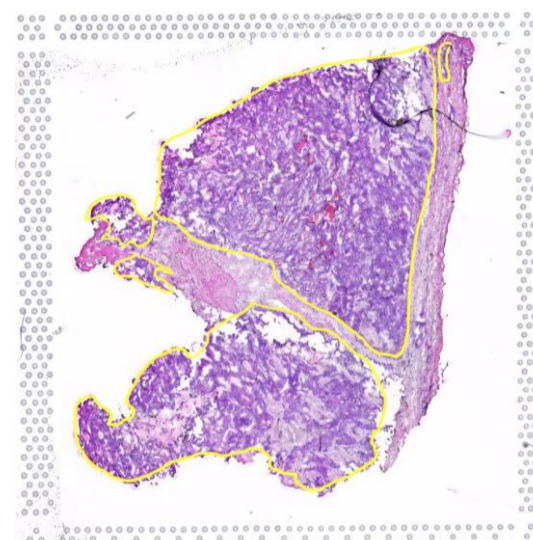

P8-N

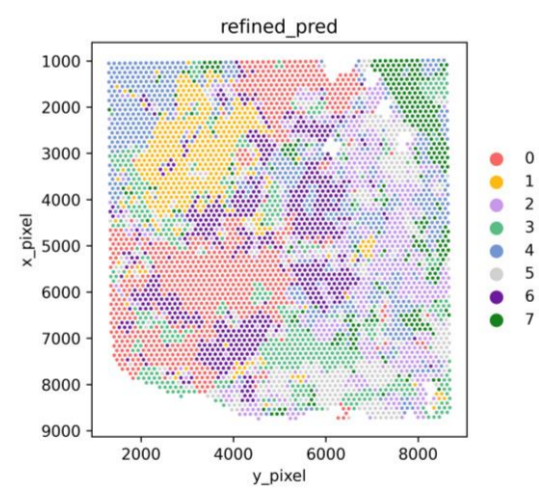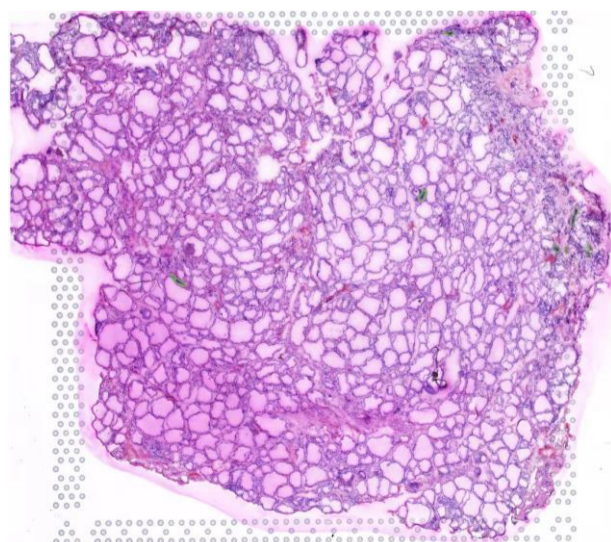

P8-T

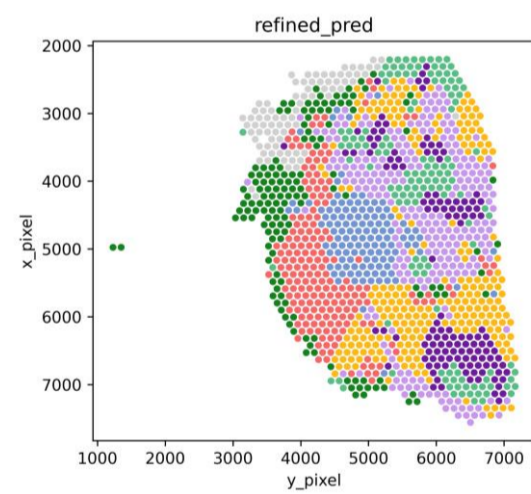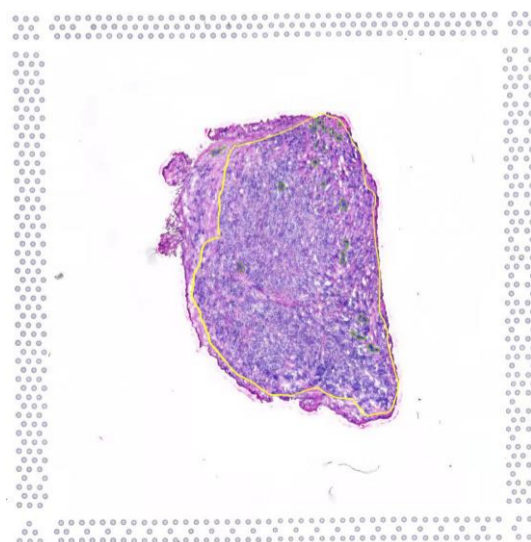

P1-L

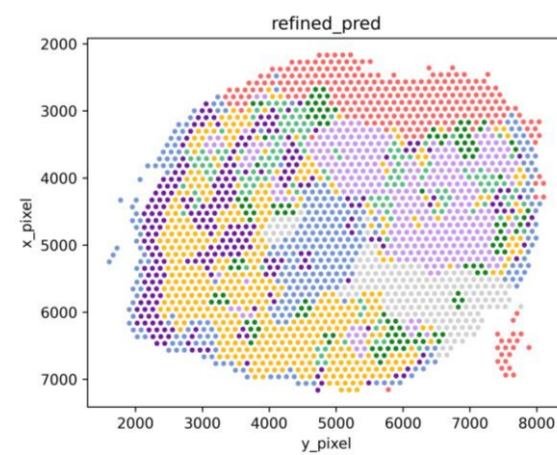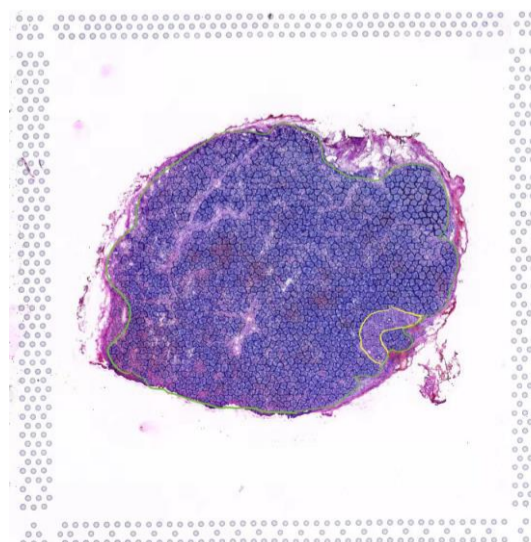

P1-T

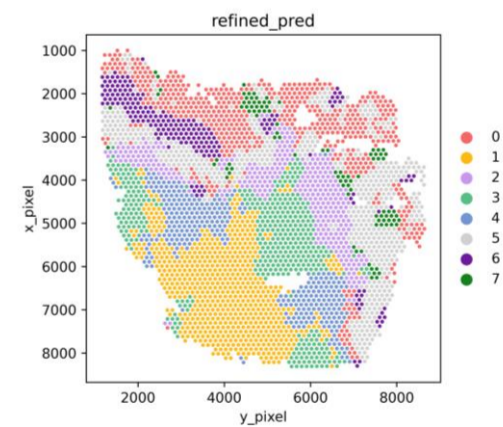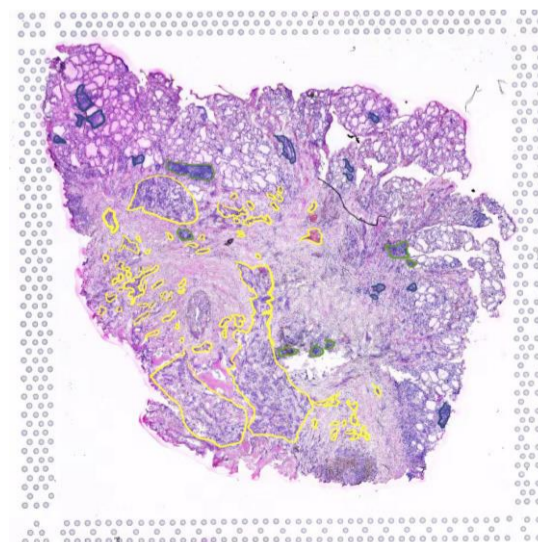

Supplement: Supplementary file 6 — Supporting information [file CTM2-15-e70172-s001.pdf]

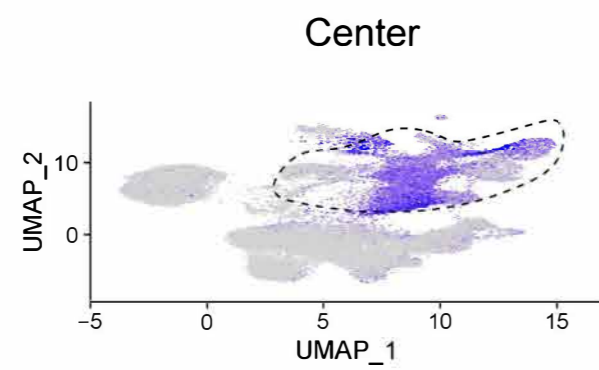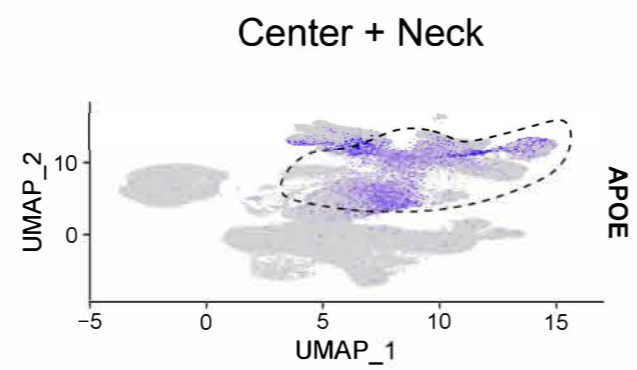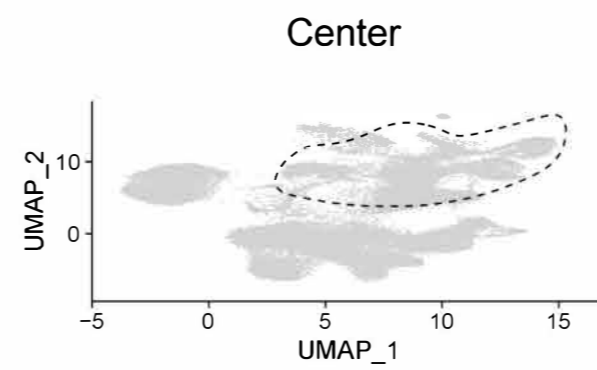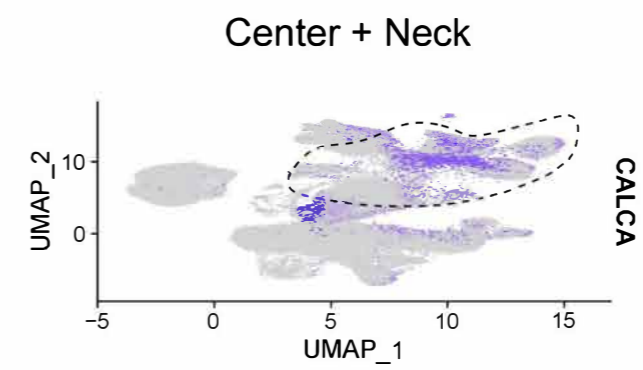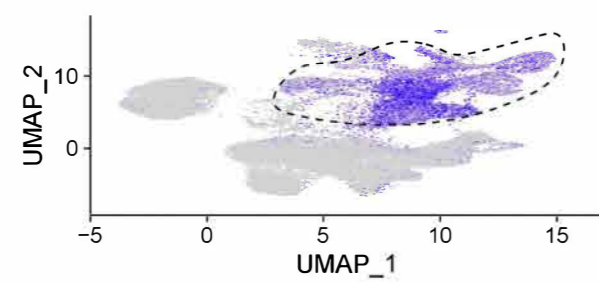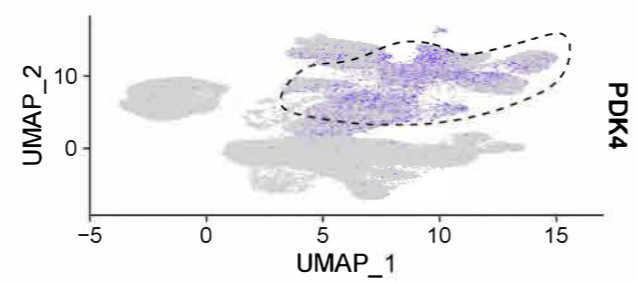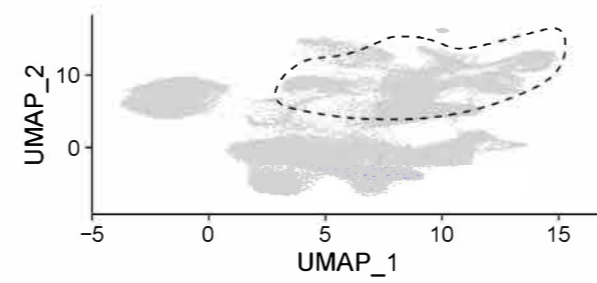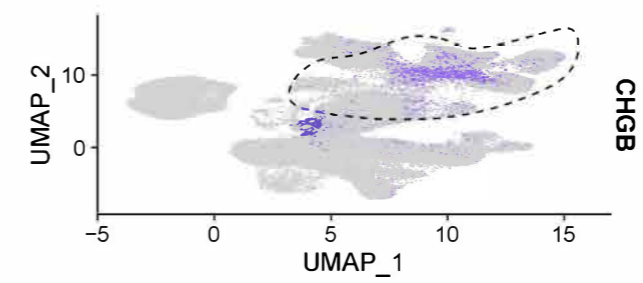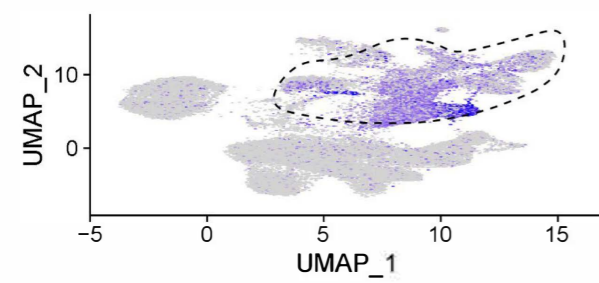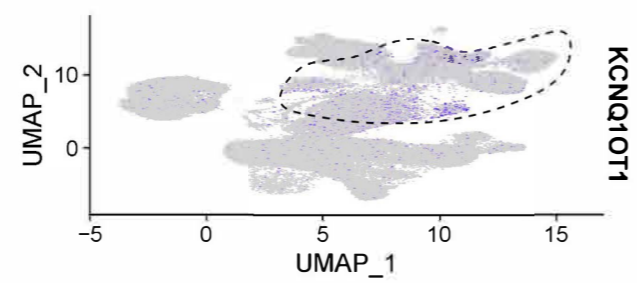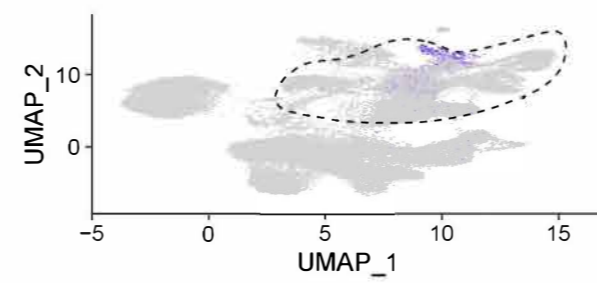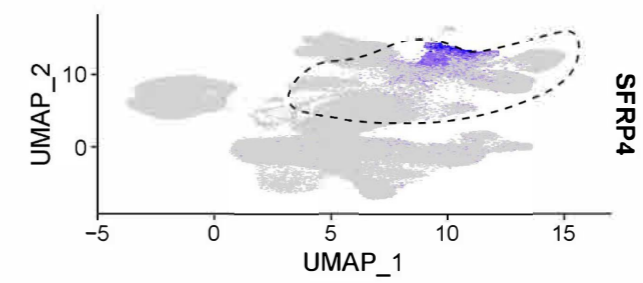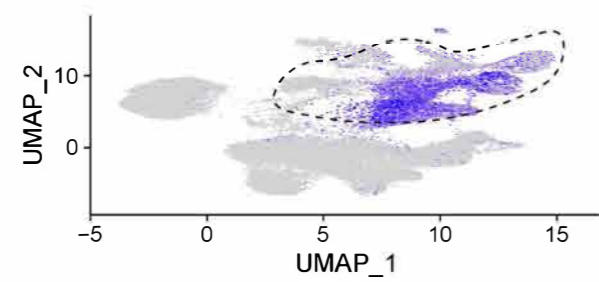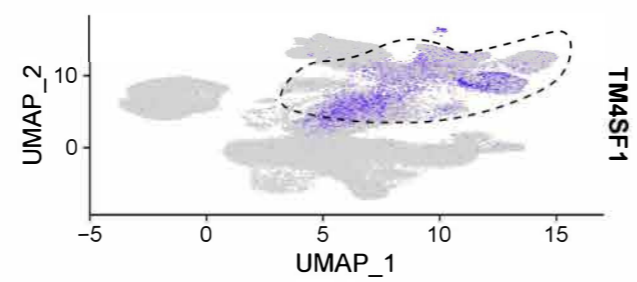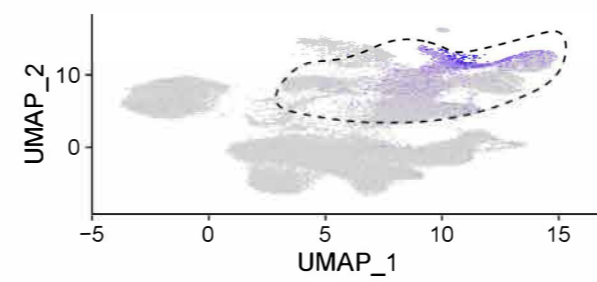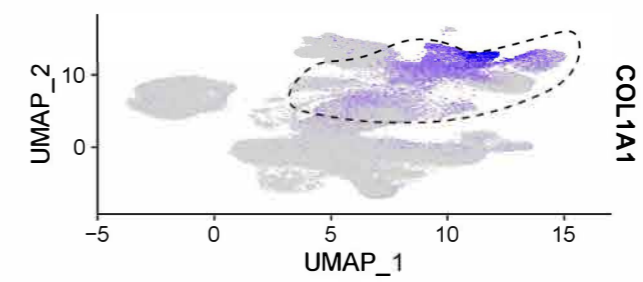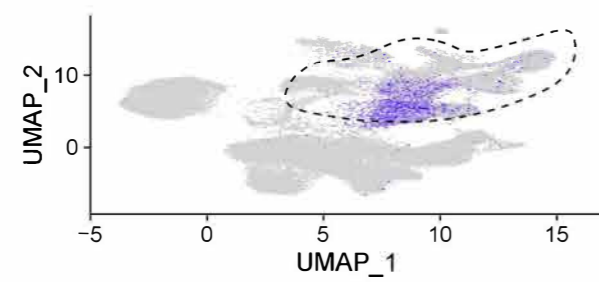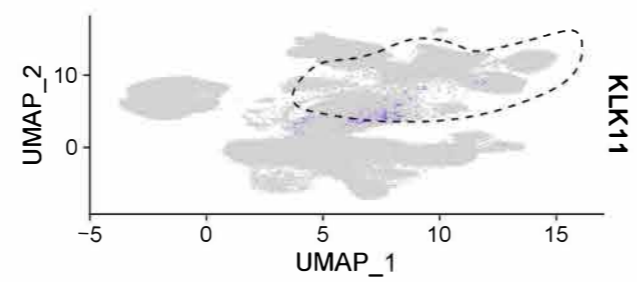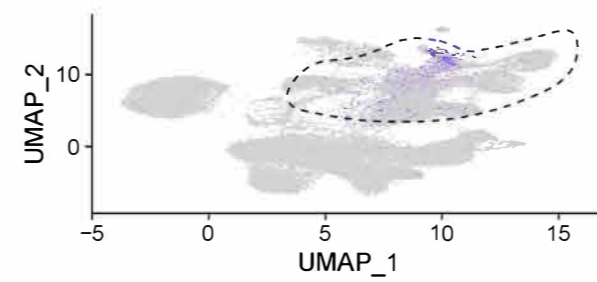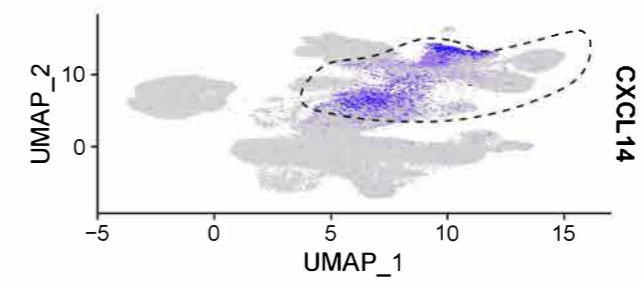

Supplement: Supplementary file 7 — Supporting information [file CTM2-15-e70172-s003.pdf]

A

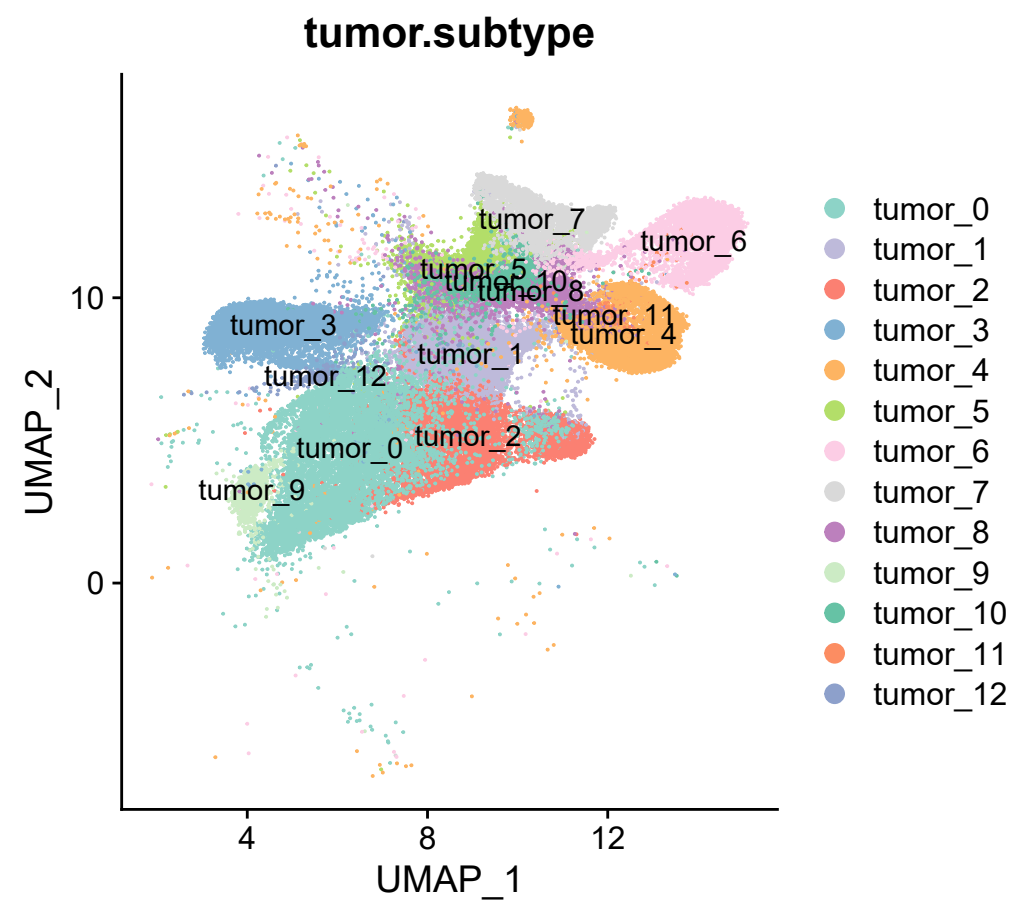

B

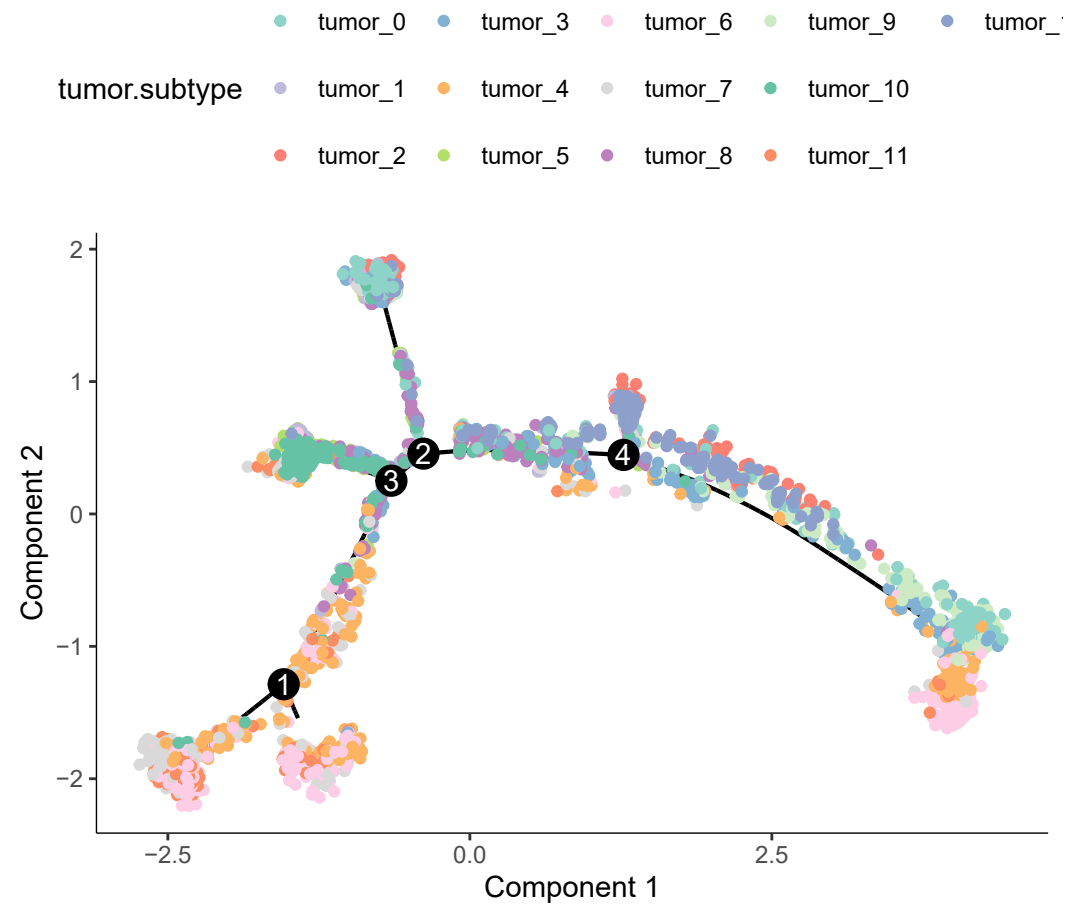

C

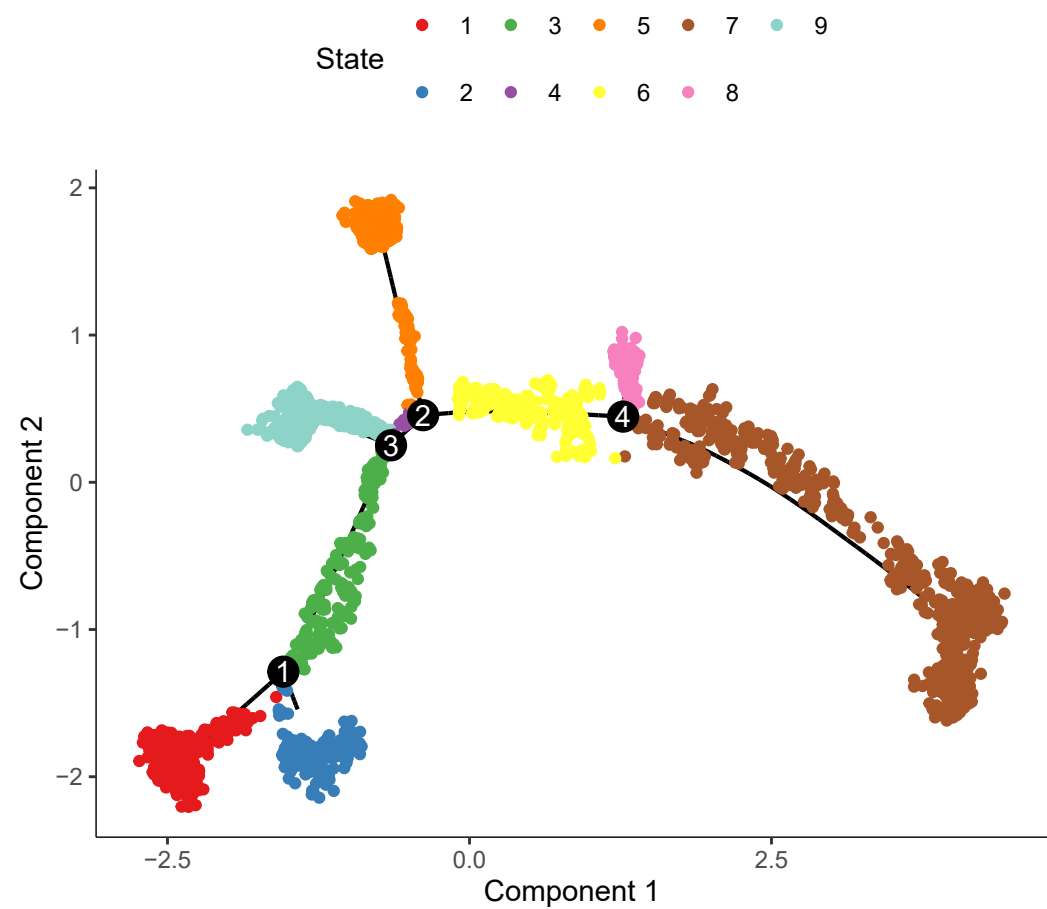

D

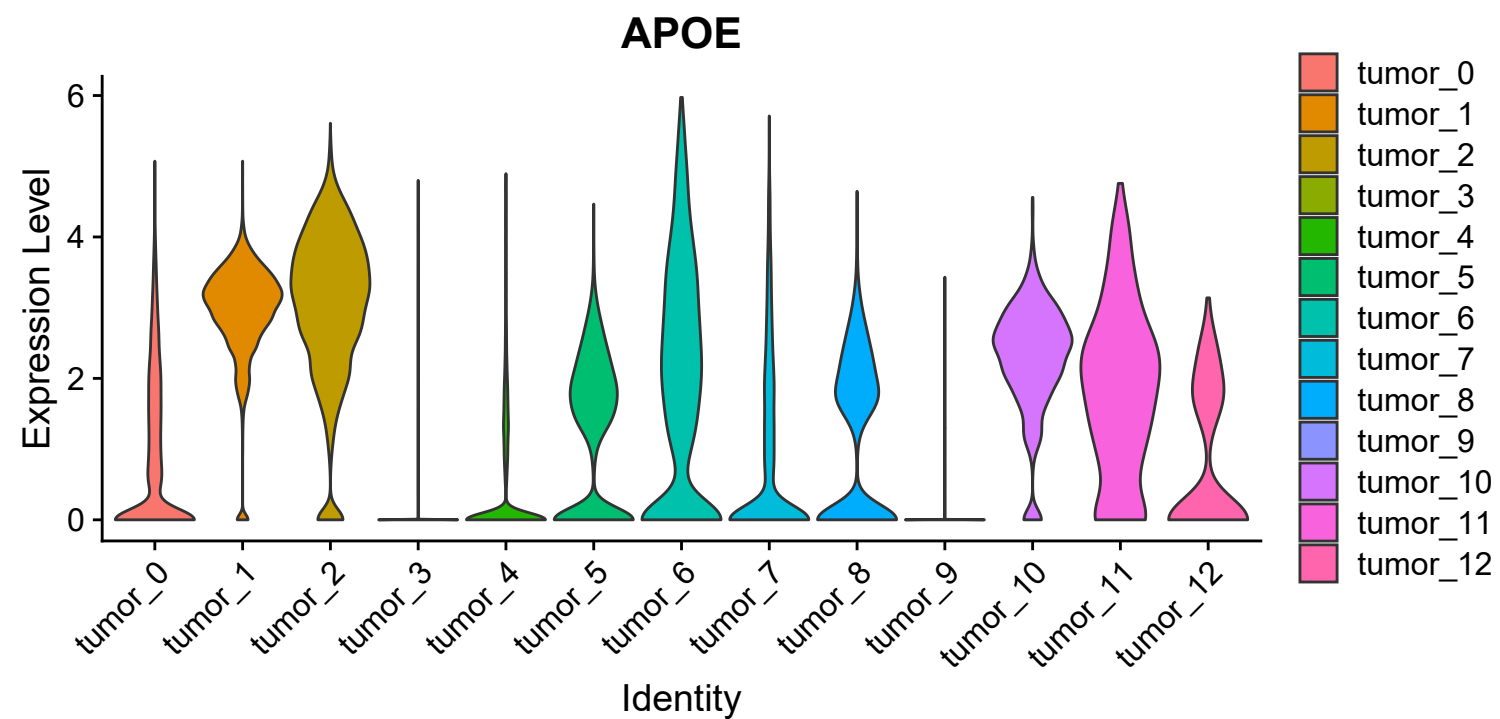

Supplement: Supplementary file 8 — Supporting information [file CTM2-15-e70172-s006.pdf]

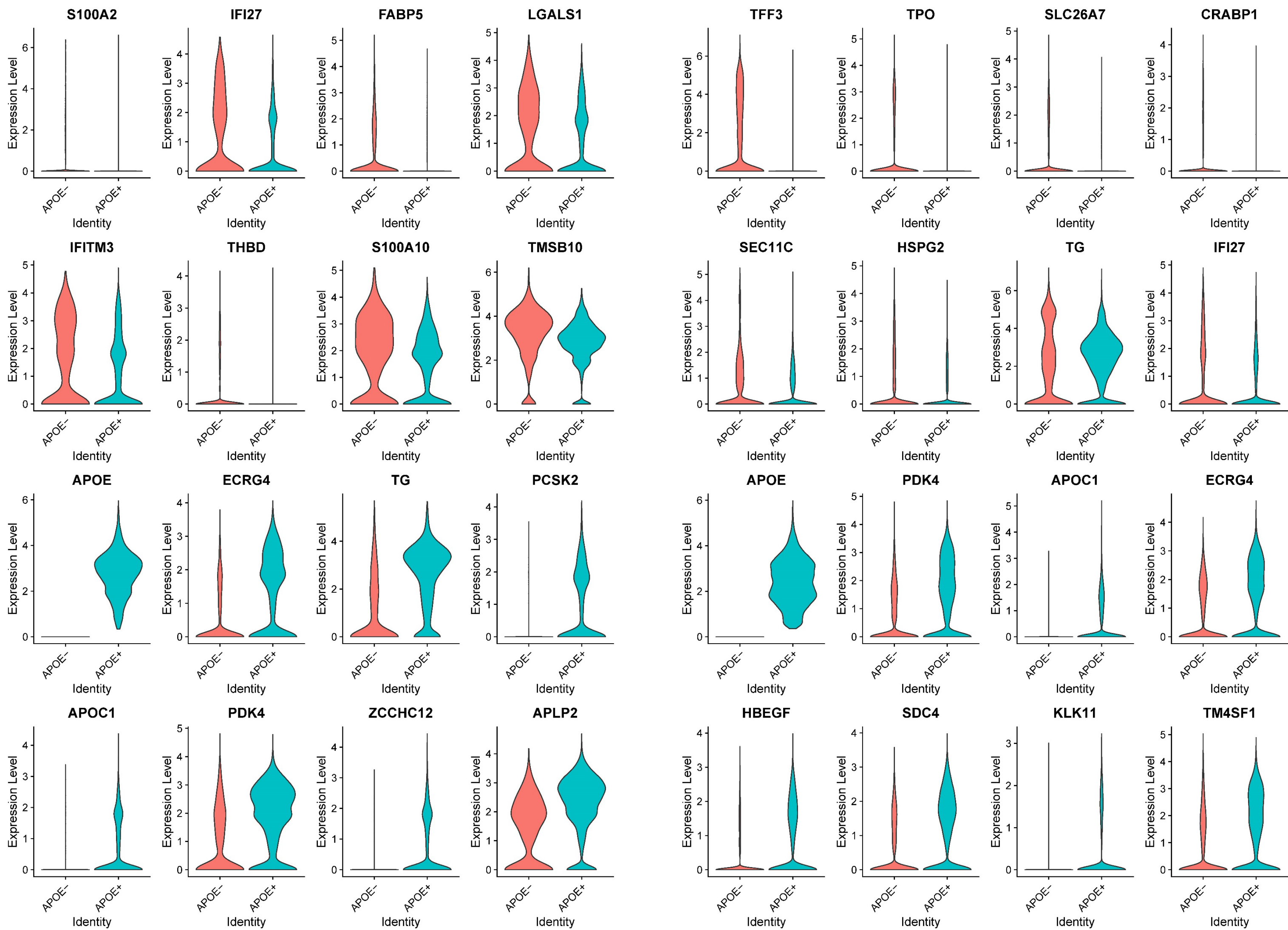

Supplement: Supplementary file 9 — Supporting information [file CTM2-15-e70172-s002.pdf]

**A**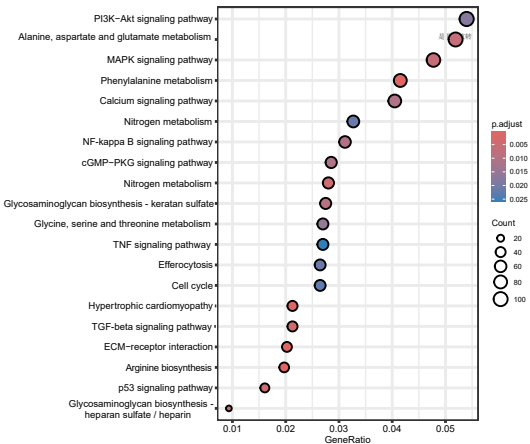**B**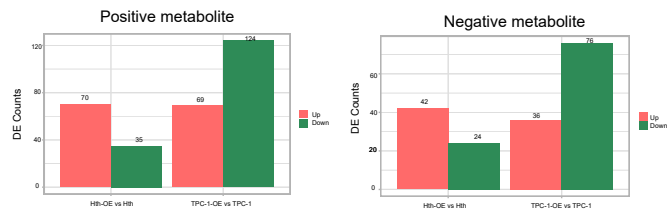**C**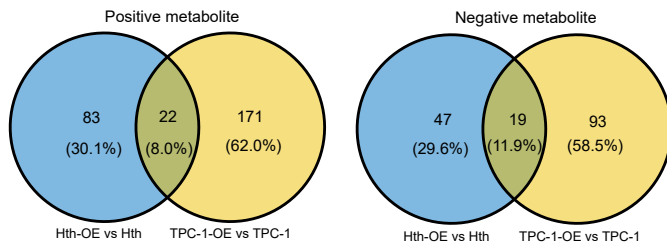**D**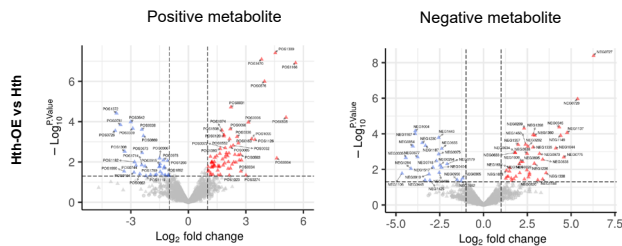**F**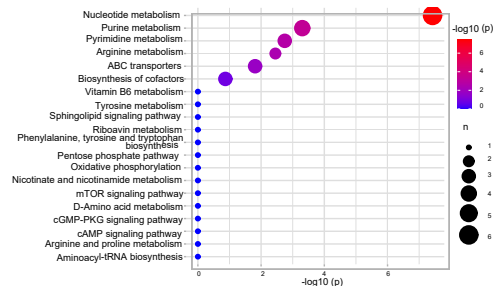**E**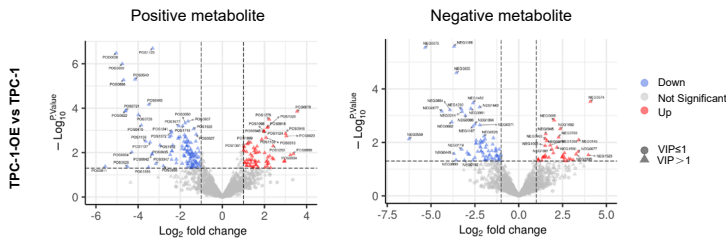**G**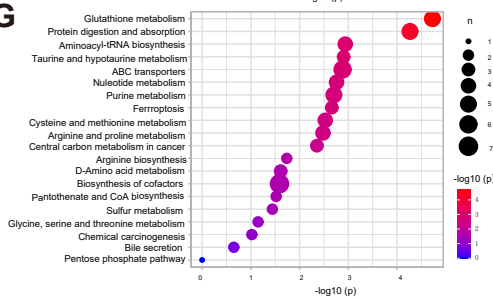

Supplement: Supplementary file 10 — Supporting information [file CTM2-15-e70172-s014.pdf]

# Proportion of cell types

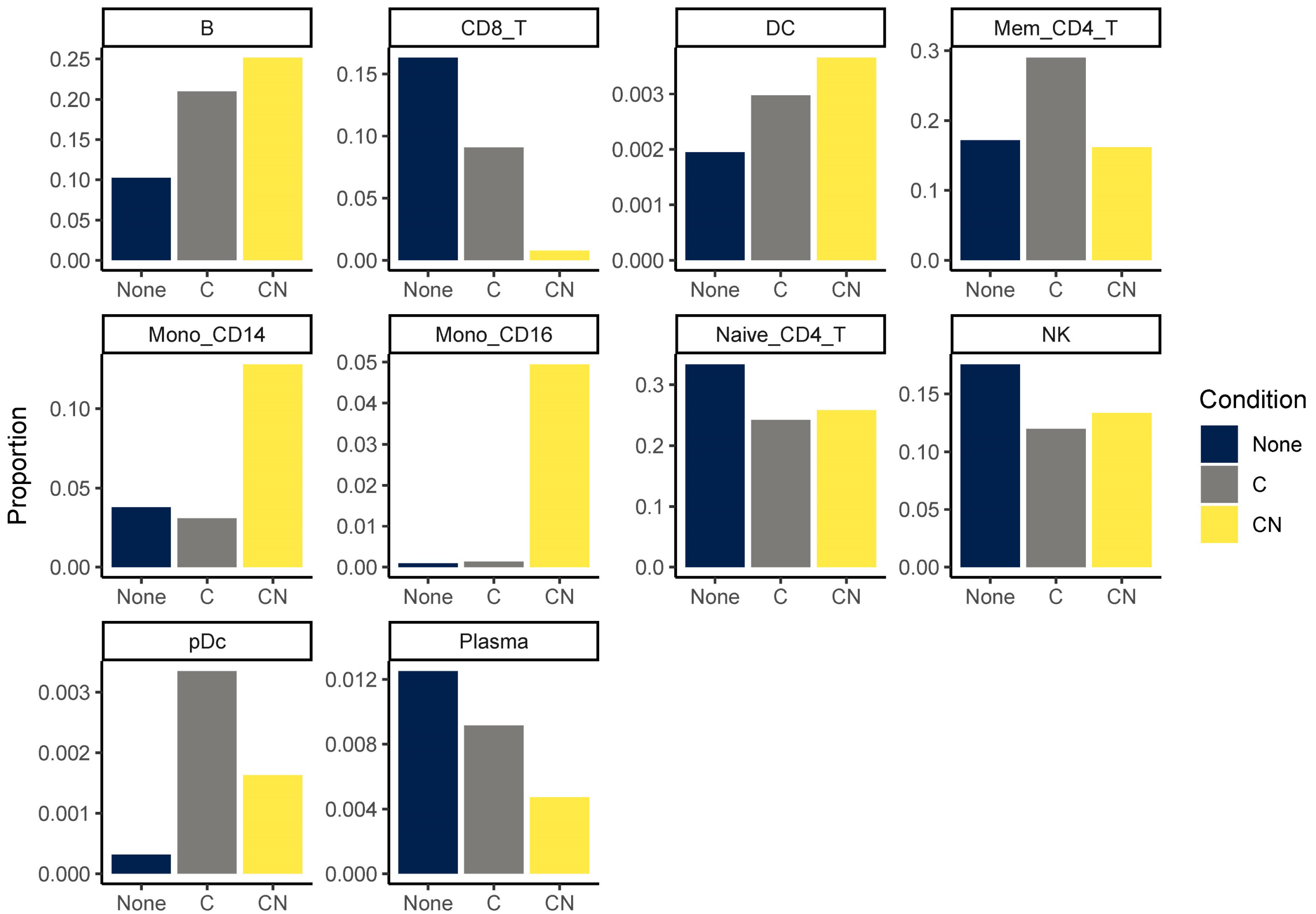

Supplement: Supplementary file 11 — Supporting information [file CTM2-15-e70172-s005.pdf]

A

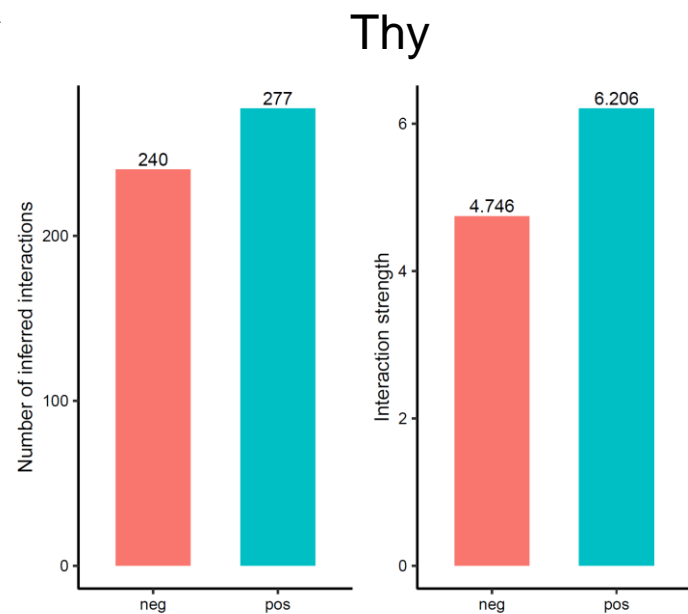

B

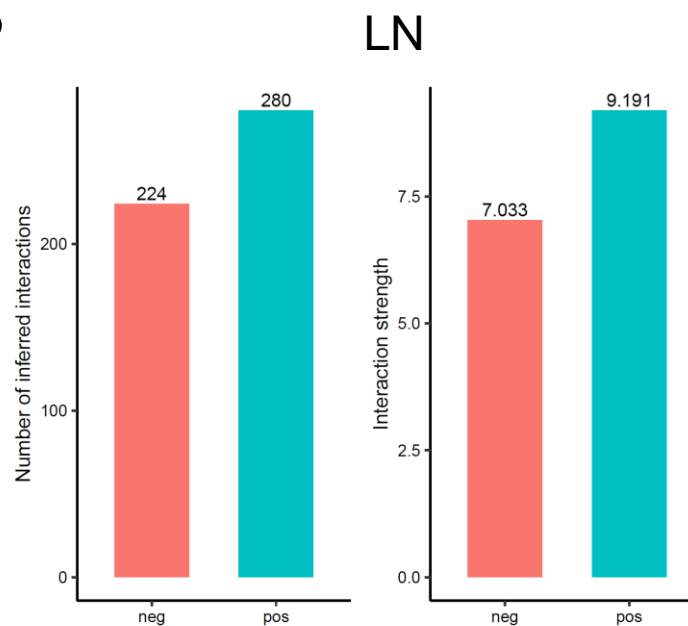

C

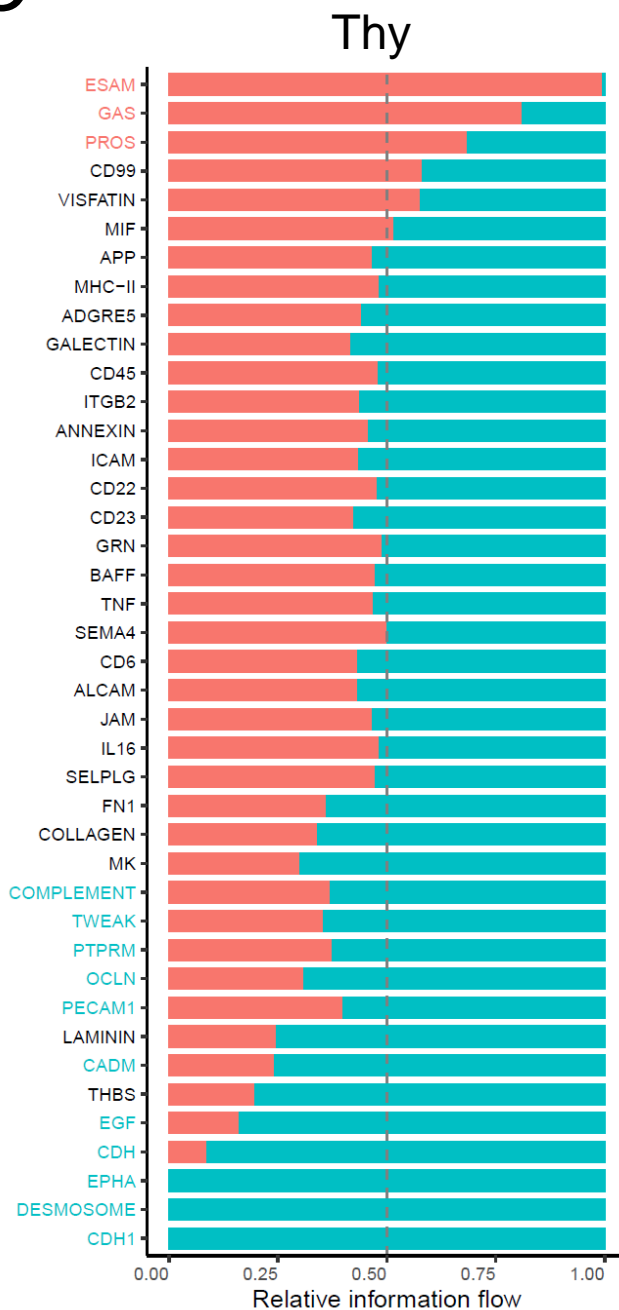

D

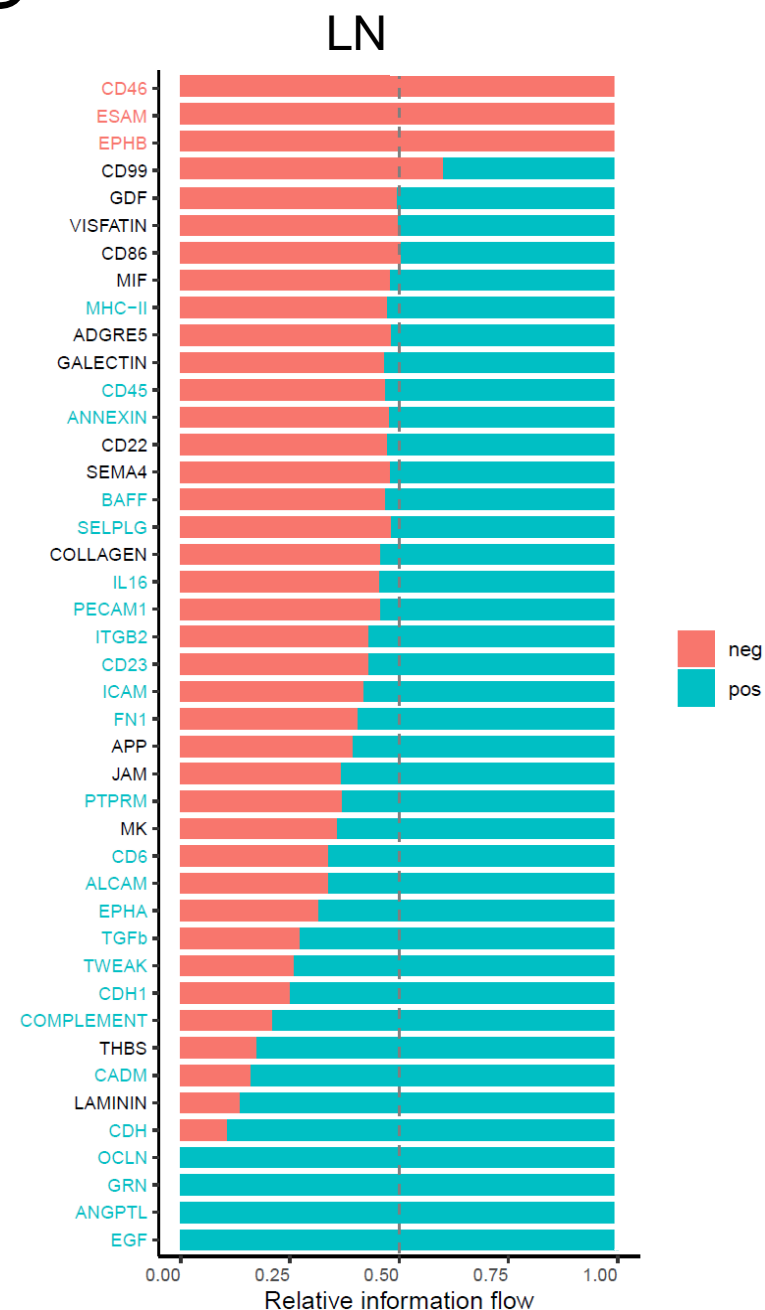

Supplement: Supplementary file 12 — Supporting information [file CTM2-15-e70172-s007.pdf]

**A**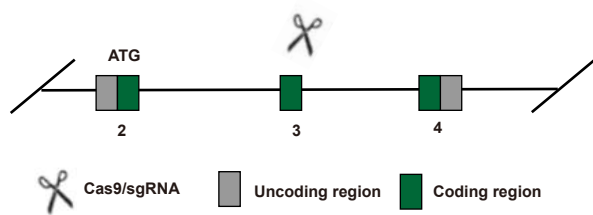**B**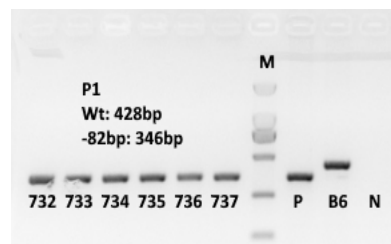**C**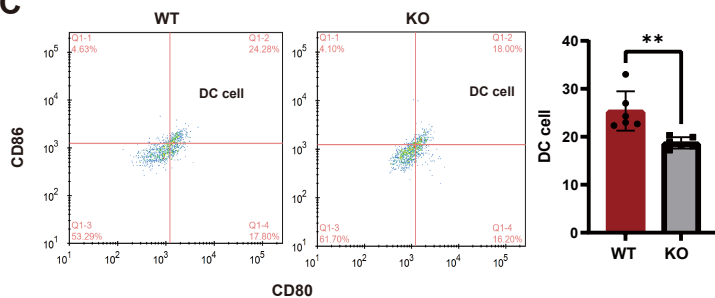**D**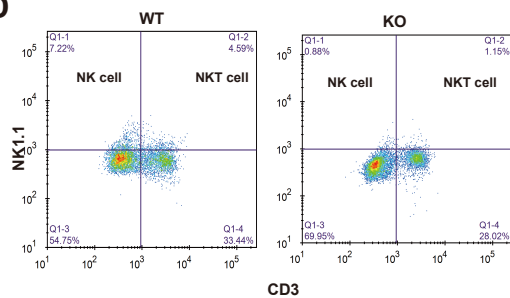**E**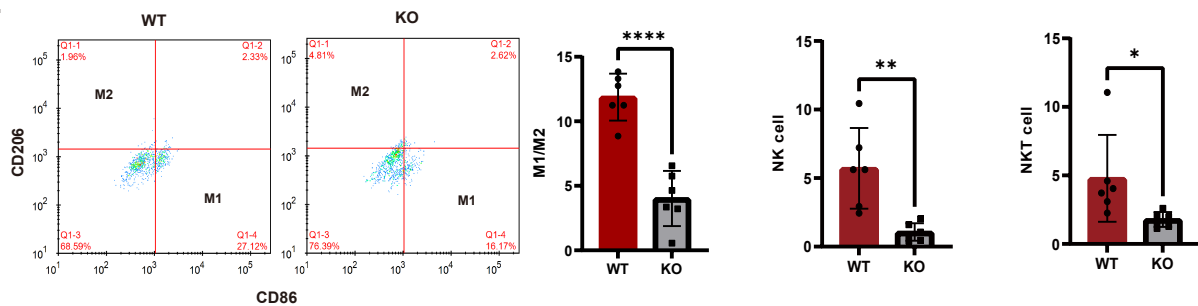**F**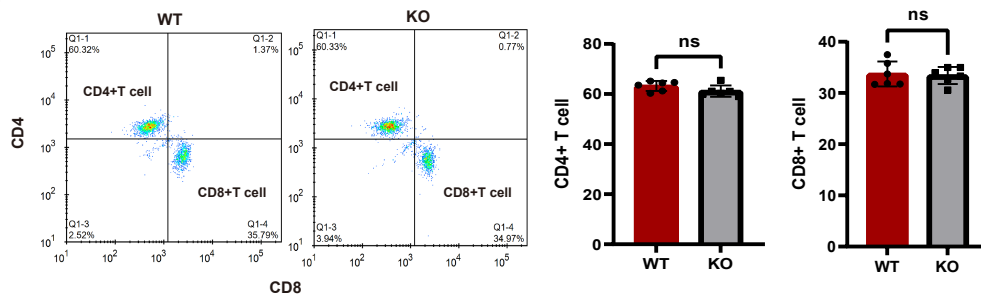

Supplement: Supplementary file 13 — Supporting information [file CTM2-15-e70172-s010.pdf]
