# Supplementary material for: Single‐cell RNA‐sequencing and spatial transcriptomic analysis reveal a distinct population of APOE − cells yielding pathological lymph node metastasis in papillary thyroid cancer
Source: Clin Transl Med. 2025 Jan 15;15(1):e70172. doi: 10.1002/ctm2.70172 (PMC11733439; doi:10.1002/ctm2.70172)
Supplement: Supplementary file 14 — Supporting information [file CTM2-15-e70172-s008.pdf]

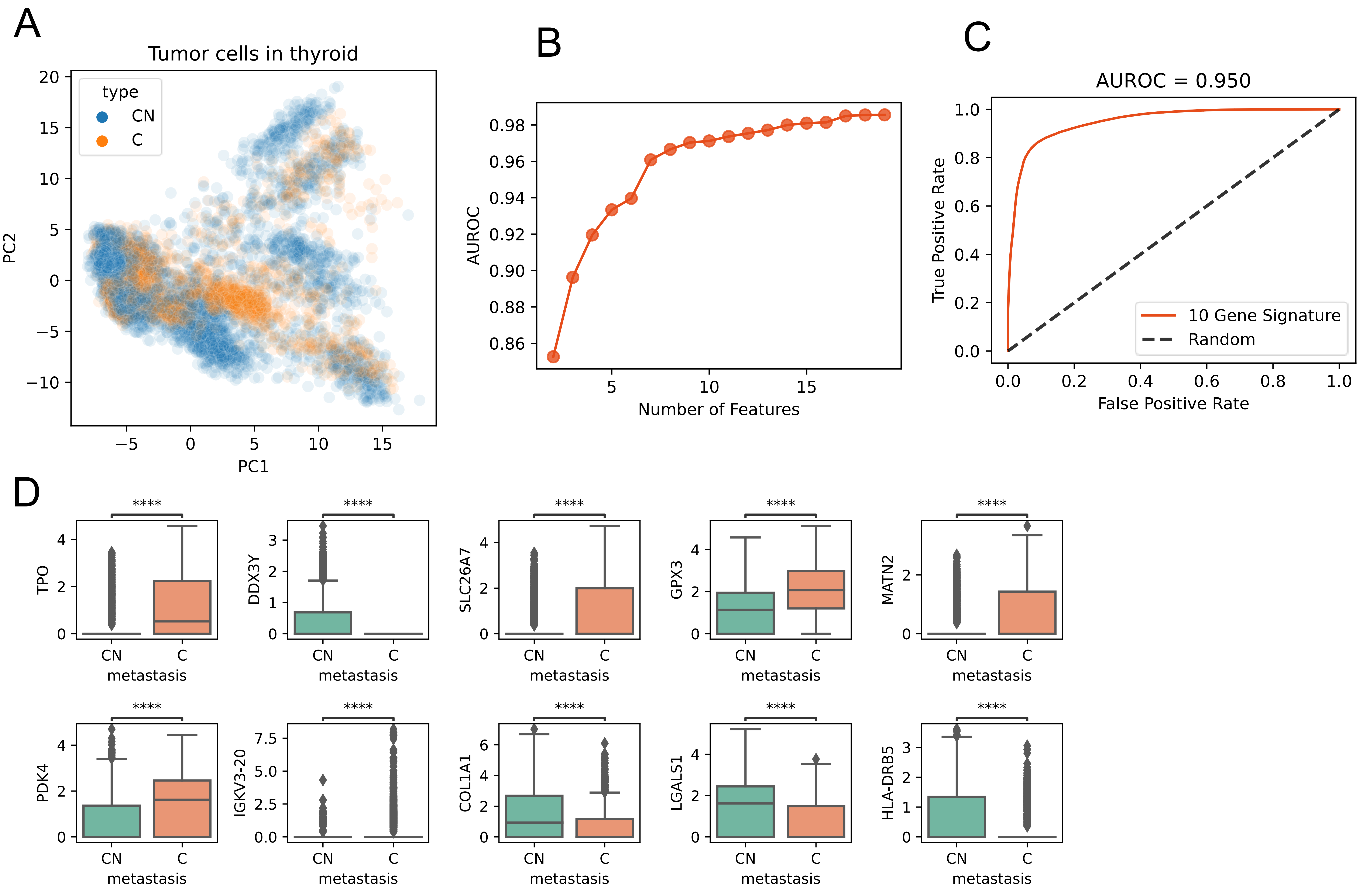

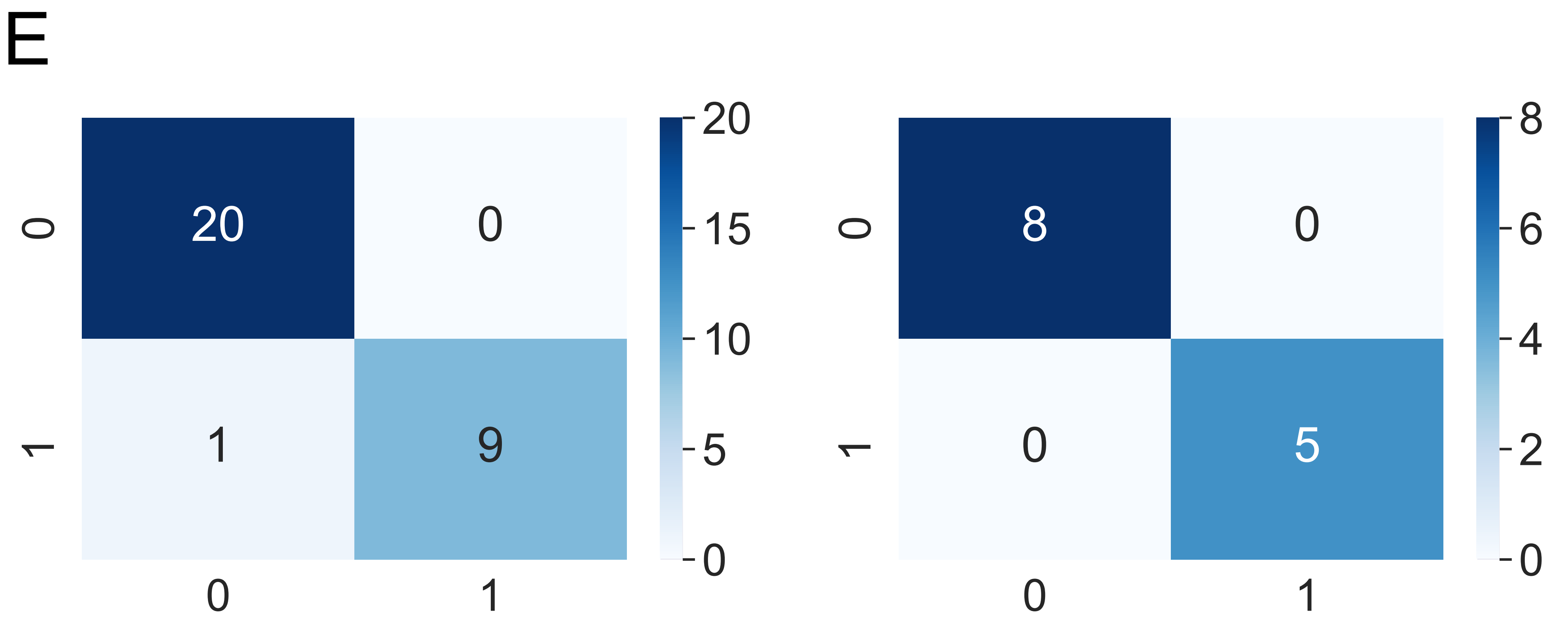

metrics	Score(sd.)
Precision	0.9883(0.0337)
Recall	0.9737(0.0756)
AUROC	1.0(0)
F1	0.9760(0.0682)
Accuracy	0.9816(0.0521)
Specificity	0.9975(0.0248)
